# Comprehensive Identification and Expression Analysis of the YTH Family of RNA-Binding Proteins in Strawberry

**DOI:** 10.3390/plants12071449

**Published:** 2023-03-25

**Authors:** Pengbo Xu, Xinyu Li, Junmiao Fan, Chong Wang, Anqi Lin, Hongli Lian

**Affiliations:** Shanghai Collaborative Innovation Center of Agri-Seeds, School of Agriculture and Biology, Shanghai Jiao Tong University, Shanghai 200240, China; xpb2005@sjtu.edu.cn (P.X.);

**Keywords:** strawberry, *YTH* domain, RNA-binding protein, expression analysis

## Abstract

Plant growth and development processes are tightly regulated at multiple levels, including transcriptional and post-transcriptional levels, and the RNA-binding protein YTH regulates gene expression during growth and development at the post-transcriptional level by regulating RNA splicing, processing, stability, and translation. We performed a systematic characterization of *YTH* genes in diploid forest strawberry and identified a total of nine *YTH* genes. With the help of phylogenetic analysis, these nine genes were found to belong to two different groups, YTHDC and YTHDF, with YTHDF being further subdivided into three subfamilies. Replication analysis showed that *YTH3* and *YTH4* are a gene pair generated by tandem repeat replication. These two genes have similarities in gene structure, number of motifs, and distribution patterns. Promoter analysis revealed the presence of multiple developmental, stress response, and hormone-response-related *cis*-elements. Analysis of available transcriptome data showed that the expression levels of most of the *YTH* genes were stable with no dramatic changes during development in different tissues. However, *YTH3* maintained high expression levels in all tissues and during fruit development, and *YTH4* was expressed at higher levels in tissues such as flowers, leaves, and seedlings, while it was significantly lower than *YTH3* in white fruits and ripening fruits with little fluctuation. Taken together, our study provides insightful and comprehensive basic information for the study of *YTH* genes in strawberry.

## 1. Introduction

Plants cannot migrate and can only passively adapt to the external environment in which they live. In this process, plants respond by regulating their own gene expression levels to induce self-physiological changes. It is well known that gene expression is influenced mainly at the transcriptional and post-transcriptional level. At the transcriptional level, epigenetic regulation, including nucleosome histone methylation and DNA methylation, is an important regulatory modality that controls the level of gene transcription [1,2]. Existing studies have shown that in addition to DNA, which can undergo multiple modifications such as methylation, RNA can also be modified in a variety of ways following transcription [3]. Among all RNA chemical modifications, m6A is the most common and abundant type of methylation modification in eukaryotic mRNA [4]. Three types of proteins play a major role in m6A modifications. The first group is methyltransferase proteins, which mainly include METTL3, METTL14, WTAP, and RBM15/RBM15B [5,6,7,8,9]; these are also known as writers. These proteins mediate the m6A methylation modification in GAC or AAC motifs in mRNA. The second class is demethylase proteins that have methylation removal effects. These are also known as erasers and include FTO and ALKBH5 [10,11,12]. These proteins result in the removal of methylation upon being recruited to mRNA. The presence of these two types of proteins makes the m6A modification of RNA a dynamic and reversible modification. The third type is RNA reading proteins, which can recognize RNA molecules with m6A modifications.

Studies on m6A methylation modifications of RNA are more numerous in animals and yeast. These studies have found that m6A modifications affect mRNA transport, translation, splicing, and degradation [13] and are involved in a variety of life processes such as spermatogenesis [14], adipogenesis [15], cell development [16], and regeneration [17]. Although investigation of m6A in plants began later, with the help of existing research methods in animals, the exploration of m6A in plants has progressed rapidly and has become a hot topic of current research. In *Arabidopsis*, the writers MTA (homolog of METTL3) and FIP37 (homolog of WTAP) are highly expressed mainly in the apical meristem, young leaves, and floral organs, affecting cell proliferation in the meristem and embryo formation [18]. The eraser ALKBH10b (homolog of ALKBH5) in *Arabidopsis* specifically regulates the flowering transition by mediating mRNA demethylation of FT, SPL3, and SPL9 [19]. Methylation of mRNAs for NCED5, AREB1, and ABAR by MTA and MTB in strawberry improves mRNA stability of NCED5 and AREB1 and promotes translation efficiency of ABAR, which ultimately promotes fruit ripening [20]. Under drought conditions, MdMTA in apple promoted m6A modification of stress-response-related genes, enhanced mRNA stability and translation efficiency of these genes, and ultimately improved drought tolerance by inducing lignin deposition and reactive oxygen species removal [21]. In tomato, the m6A demethylase SlALKBH2 decreases the level of m6A modification on SlDML2 and promotes its mRNA stability, thus promoting fruit ripening [22].

m6A modification of RNA exerts its regulatory effect in two main ways. One is that the presence of the m6A modification can change the spatial structure of RNA molecules, which in turn affects their function. The other is that m6A modifications can recruit specific proteins or complexes to mediate the function of RNA [4]. m6A reader proteins are one of the specific proteins that recognize such modifications. They usually contain a conserved YT521-B homologs structural domain and are therefore also known as YTH proteins [23]. The studies in mammals have identified five YTH proteins, namely YTHDC1 (YTH domain-containing protein 1), YTHDC2 (YTH domain-containing protein 2), and YTHDF (YTH domain-containing family protein), which includes YTHDF1/2/3. The similarity between these three YTHDF proteins is high, and the YTH structural domain is at the C-terminus [4,23]. The sequence of YTHDC1 and YTHDC2 proteins has no significant similarity except for the conserved YTH domain, which is located in the middle of the sequence of YTHDC1, while the YTH domain of YTHDC2 is located at the C-terminus. However, in addition to the YTH domain, YTHDC2 also contains several structural domains including ANK, helicase N, and helicase C domains. In addition to variations in protein similarity, there are also differences in the localization of YTH proteins; YTHDF and YTHDC2 are mainly localized in the cytoplasm, whereas YTHDC1 is mainly located in the nucleus [23,24]. Unlike in animals, the variety and numbers of YTH proteins in plants are more complicated. By a homology matching search, 13 *YTH* genes were found in the *Arabidopsis* [25]. Mutations in the m6A reader protein ECT2/3/4 in *Arabidopsis* cause changes in leaf morphology and delay leaf development [26]. Recent studies have shown that CPSF30-L, a homolog of YTHDC in *Arabidopsis*, can regulate mRNA poly(A) site selection by recognizing m6A modifications, which in turn affects the expression of nitrate-signaling-related genes and regulates plant nitrogen uptake and assimilation [27]. In addition, Hou et al. found that CPSF30-L could regulate selective polyadenylation by recognizing the FUE element of the poly(A) site signaling sequence on mRNA to influence flowering time and response to ABA in plants [28]. There are fewer studies on YTH proteins in horticultural plants. Studies in apple have shown that YTH proteins MhYTP1 and MhYTP2 enhance tolerance to biotic and abiotic stresses [29]. Taken together, YTH proteins play an important role in several biological processes including growth and development, nutrient uptake, and stress responses.

The m6A modification performs diverse functions through binding to specific proteins. In this process, YTH proteins are the key proteins that recognize m6A modifications. Most of the studies on YTH genes and proteins in plants have been focused on the model plant *Arabidopsis* and rice, and there is very limited information on relevant studies in horticultural plants. Strawberry is a model plant for studying berry horticultural plants, but so far, there are no clear studies on YTH genes or proteins. To study the role of YTH proteins in strawberry, the first thing to do is to accurately and comprehensively identify how many YTH proteins are present in strawberry and get the basic characteristics of these YTH proteins. In the present study, we performed a comprehensive analysis of *YTH* genes in strawberry including identification of *FvYTH* genes, chromosomal location, phylogenetic analysis, conserved motifs, gene structure, and *cis-*acting regulatory elements in the promoter regions. In addition, we also investigated the expression patterns of *FvYTH* genes in different tissues and under different development stages of fruits. Our results provide some clues for the functional elucidation of *FvYTH* genes in the growth and development of strawberry. This was a fundamental but necessary study.

## 2. Results

### 2.1. Genome-Wide Identification and Characterization of FvYTHs in Strawberry

To identify the YTH proteins in strawberry, we searched the protein database of strawberry with the HMM module of the YTH structural domain and 13 *Arabidopsis* YTH proteins, respectively. Then after removing redundant sequences, nine YTH proteins were obtained (Supplementary Table S1) and were named as FvYTH1 to FvYTH9 according to the order of their gene IDs (Table 1). The nine protein sequences were further validated by Pfam, InterPro, and SMART. The results showed that all sequences have the typical YTH domain (PF04146), which indicates that they belong to the *YTH* gene family. Among these nine YTH proteins, FvYTH7 has the shortest protein length of 355 amino acids and also has the smallest molecular weight of 40.46 kDa. On the other hand, FvYTH3 has the longest length of 773 amino acids and the largest molecular weight of 84.98 kDa. The isoelectric point ranges from 5.12 (FvYTH5) to 8.53 (FvYTH3) (Table 1). In addition, the GRAVY (Grand Average of Hydropathicity) values of YTH proteins were all below zero, implying that they are all hydrophilic proteins. Among them, FvYTH1 was the most hydrophilic with a GRAVY value of −0.964, while FvYTH6 was the least hydrophilic with a GRAVY value of −0.593. The protein stability prediction results showed that the stability indexes of FvYTH3/4/6/8 were less than 40, implying that their proteins were unstable. Conversely, the stability indexes of the remaining five FvYTH proteins were all above 40, implying that they have a certain level of stability. Subcellular localization predictions suggested that all FvYTH proteins in strawberry are localized in the nucleus (Supplementary Table S2).

### 2.2. Chromosome Localization and Synteny Relationships of FvYTH Genes

**Figure 1:**
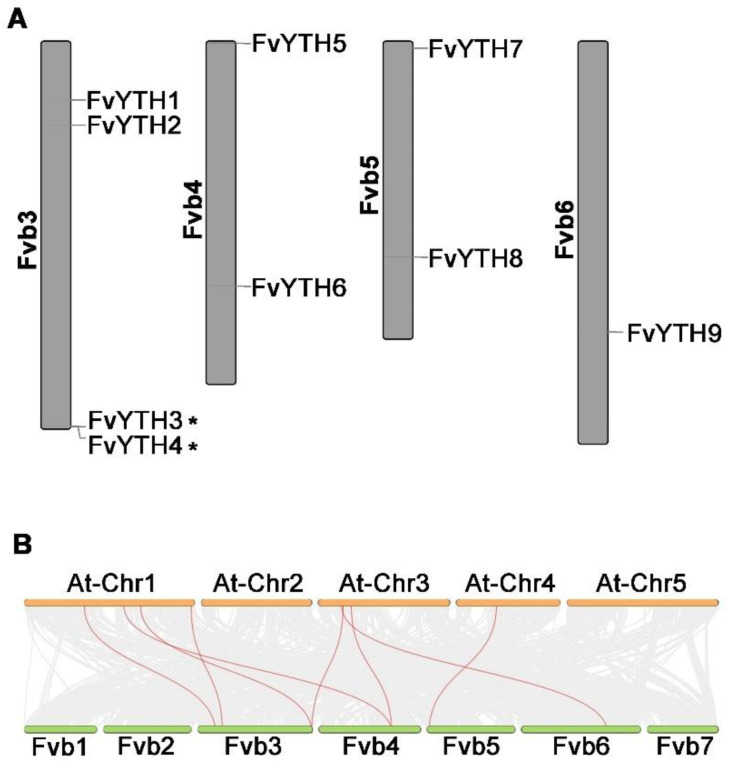
The locations and synteny analysis of strawberry *FvYTH* genes. (**A**) The locations of *FvYTH* genes in forest strawberry chromosomes. The chromosome number is indicated at the left of each chromosome. Gene pairs from tandem duplications are marked with *. (**B**) The synteny genes between strawberry and *Arabidopsis*. The red lines indicate the synteny gene pairs of *YTH* genes.

To determine the localization of the nine *FvYTH* genes on the chromosomes, we mapped the chromosomal localization of the *FvYTH* genes. Figure 1A shows that these nine *FvYTH* genes are distributed on chromosomes three to six, and no *FvYTH* genes are located on chromosomes one, two, or seven. Four of the genes are located on chromosome three, chromosomes four and five each contain two genes, and chromosome six has only one *FvYTH* gene. Two genes (*FvYTH3* and *FvYTH4*) are clustered into one tandem duplication event region on chromosome three (Figure 1A). No segment duplication events of *FvYTH* were found. Furthermore, we performed comparative syntenic maps between strawberry and *Arabidopsis thaliana*. It was found that the six *FvYTH* genes, including *FvYTH1/2/3/6/7/9*, in strawberry formed a total of eight collinear gene pairs with *Arabidopsis* (Figure 1B and Supplementary Table S3). Among them, *FvYTH3* and *FvYTH6* could form syntenic gene pairs with two *YTH* genes in *Arabidopsis*. No syntenic gene pairs were found in *Arabidopsis* for *FvYTH4/5/8*. The presence of these evolutionarily conserved collinear gene pairs suggests that the function of these genes may also be somewhat conserved among species.

### 2.3. Gene Structure, Conserved Domain, and Motif Analysis of FvYTHs

**Figure 2:**
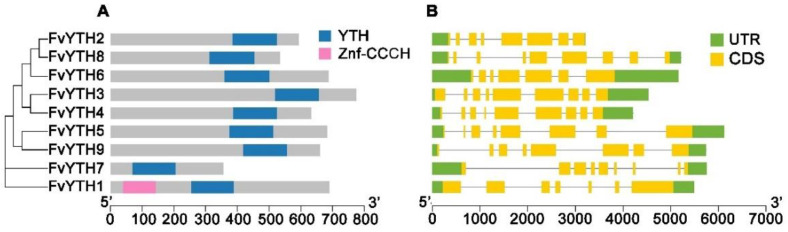
Gene structures and conserved domains of FvYTH proteins. (**A**) Conserved domains in FvYTH proteins. Pink box represents the Znf-CCCH domain, and blue boxes represent the YTH domain. (**B**) Exon/intron organizations of *FvYTH* genes. Yellow boxes represent exons, and gray lines represent introns. The upstream/downstream regions of *FvYTH* genes are indicated in green boxes.

**Figure 3:**
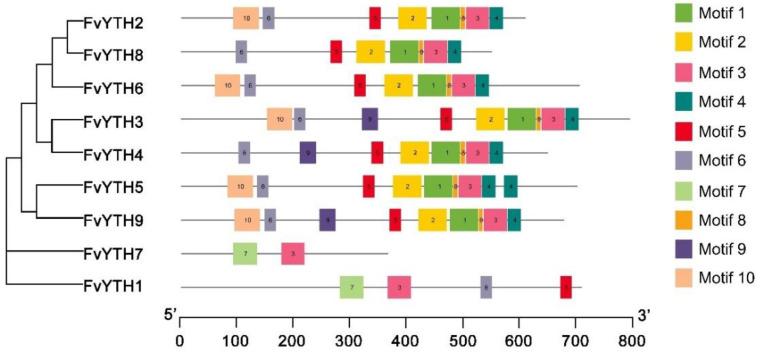
Distributions of conserved motifs in FvYTH proteins. Ten putative motifs are indicated in different colored boxes.

We aligned the mRNA sequences of *FvYTHs* with the genome sequence of strawberry and generated exon–intron distribution maps of these nine *FvYTH* genes using TBtools. As shown in Figure 2B, all of these *FvYTH* genes contain six to nine introns, and genes clustered together have a similar distribution of gene structures. Additionally, analysis of the 10 most conserved motifs (Supplementary Figure S1) in FvYTH proteins by the MEME tool revealed that seven motifs are arranged in the same order, i.e., motif 6, motif 5, motif 2, motif 1, motif 8, motif 3, and motif 4 appear in FvYTH2 to FvYTH9, with the exception of FvYTH1 and FvYTH7. FvYTH7 contains the fewest motifs, as it contains only motif 3 and motif 7, which also appear in FvYTH1 (Figure 3). Taken together, these results indicated that the patterns of distribution of exons/introns and motifs were similar among the members within each group in the phylogenetic tree. 

Analysis of the conserved domains showed that although all nine proteins contained a conserved YTH domain, their positions were different. The YTH domain of FvYTH1 and FvYTH7 were located in the middle, while the YTH domains of the remaining seven proteins were located at the C-terminal position. In addition, the SMART and InterPro databases were searched for additional known domains that potentially exist in YTHs proteins, and these searches identified a conserved CCCH-type zinc finger (Znf-CCCH) domain in FvYTH1 proteins (Figure 2A).

### 2.4. Phylogenetic Analysis of YTH Proteins in Strawberry

**Figure 4:**
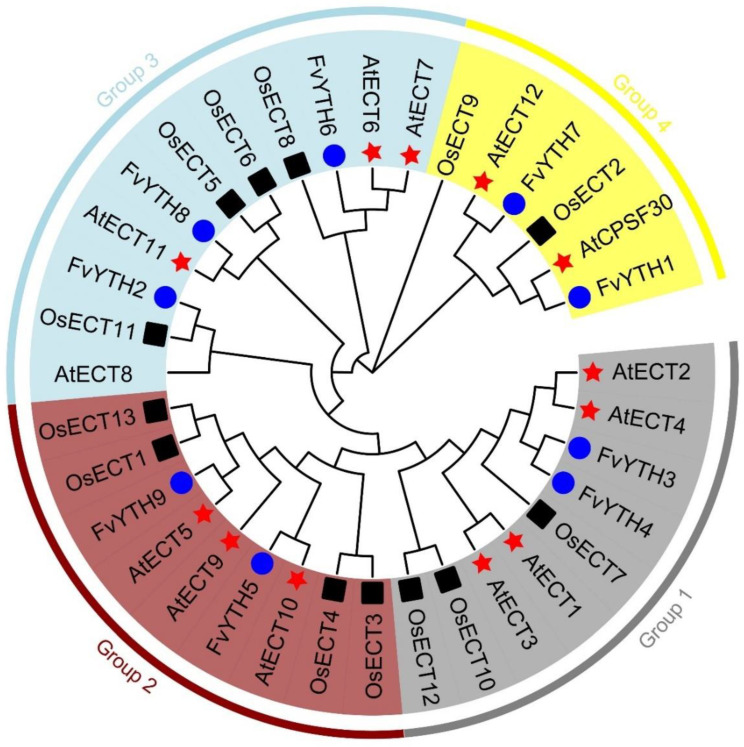
Phylogenetic analysis of YTHs in strawberry, rice, and *Arabidopsis*. The sequences of YTH domain from strawberry, rice, and *Arabidopsis* were used to construct a phylogenetic tree by NJ method, with 1000 bootstrap replications. Red stars represent *Arabidopsis*, blue circles represent rice, and black boxes represent strawberry.

In order to evaluate the evolutionary relationships of YTH proteins, we constructed phylogenetic trees by selecting YTH domain sequences of YTH proteins from *Arabidopsis*, rice, and strawberry (Supplementary Table S4). The results showed that these YTH proteins can be divided into four groups (Figure 4), among which groups one to three have only one conserved YTH domain and no other conserved domains (Figure 2A). Although FvYTH7 and FvYTH1 are evolutionarily related, both being in group four, the FvYTH1 protein contains two conserved domains, YTH and Znf-CCCH, while the FvYTH7 protein contains only one YTH domain (Figure 2A). In humans, there are five YTH proteins, including YTHDC1, YTHDC2, YTHDF1, YTHDF2, and YTHDF3, which are divided into two subfamilies: YTHDF and YTHDC. To further classify which subgroup these nine YTH proteins of strawberry belong to, we constructed a phylogenetic tree by putting together nine YTH proteins from strawberry and five YTH proteins from humans, and the results showed that FvYTH1 and FvYTH7 belong to the YTHDC subgroup, while the remaining seven YTH proteins belong to the YTHDF subgroup (Supplementary Figure S2). The results of multiple sequence alignment showed that in the YTHDF subgroup, 54 amino acid residues were identical, and 20 residue positions were found to have greater than 75% homology (Supplementary Figure S3, amino acids in black and gray background). In the YTHDC subgroup, 42 residues were fully conserved, and 30 amino acids sites were highly conserved (Supplementary Figure S4, amino acids in black and gray background). It has been shown that in the m6A–YTH complex, the methylated RNA is mainly wrapped by the positively charged groove formed by WWW/L in the YTH domain. These three amino acids are very conserved in FvYTH2 to FvYTH9, while in FvYTH1, the third position of tryptophan or leucine is replaced by tyrosine (Supplementary Figures S3 and S4, amino acids indicated by asterisks).

### 2.5. Cis-Acting Regulatory Elements in Promoter Regions of FvYTH Genes

**Figure 5:**
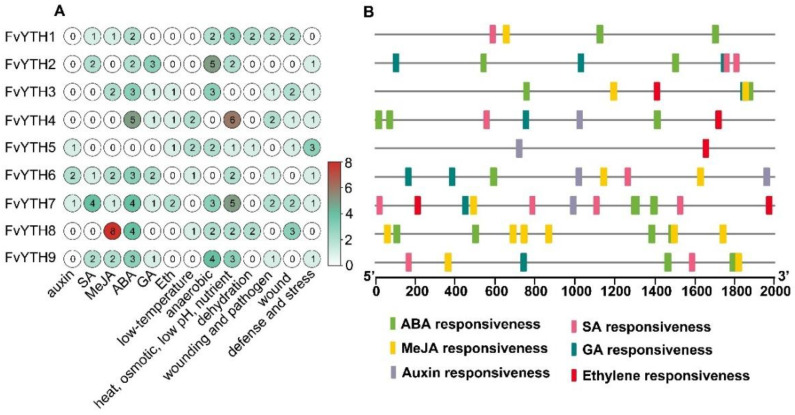
The *cis*-elements analysis of promoters in *FvYTH* genes. The 2 kb DNA fragment upstream of the initiation codon of each *FvYTH* gene was analyzed using the online software PlantCARE. (**A**) The number of *cis*-elements related to hormones and stress in each *FvYTH* promoter. (**B**) The distribution of hormone-response elements in the *FvYTH* gene promoters.

To analyze biological functions FvYTH proteins may be involved in, we searched 2000 bp upstream of the start codon of each *FvYTH* gene for analysis of *cis-*acting elements. The results showed that the promoters of *FvYTH* genes usually contain a high number of *cis*-elements related to hormone, stress, and light responses (Supplementary Table S5). The promoters of *FvYTH7* contained six kinds of hormone-response elements including ABA-, GA-, SA-, Eth-, MeJA-, and auxin-response elements. *FvYTH6* contained response elements for five types of hormones other than ethylene on its promoter, and the auxin-response element was absent in the promoter of *FvYTH9*. The *FvYTH5* promoter possessed the fewest hormone-response elements, containing only one ethylene-response element and one auxin-response element (Figure 5A,B). However, a variety of stress-related *cis*-elements that respond to external environmental stresses were found in the upstream regions of *FvYTH5* promoters, such as defense- and stress-responsive elements (TC-rich repeats), an anaerobic-induction element (ARE), a wound-response element, a dehydration-responsive element, a low-temperature-responsive element (LTR), and *cis*-elements related to heat, osmotic stress, low pH, and nutrient starvation (Figure 5A and Supplementary Table S5). The promoter of *FvYTH4* contained a higher number of ABA-response elements and *cis*-elements related to heat shock, osmotic stress, low pH, and nutrient starvation than other *FvYTH* gene promoters. The *FvYTH8* promoter contains as many as eight elements involved in the jasmonate response (Figure 5A,B). The *FvYTH2* promoter contains the highest number of anaerobic-related response elements compared to other *FvYTH* gene promoters (Figure 5A).

The *FvYTH* gene promoters contained a smaller number of development-related response elements compared to the number of hormone- and stress-related response elements. Each of the *FvYTH6/7/8* promoters has only one CAT-box, which was related to meristem expression. While *FvYTH4* and *FvYTH9* promoters contain no development-related response elements (Supplementary Table S5), the remaining four *FvYTH* gene promoters contained three to five *cis*-elements related to development- and tissue-specific expression, such as the *cis*-elements involved in meristem expression (CAT-box and CCGTCC-box), palisade mesophyll cells, meristem-specific activation, circadian control (circadian), zein metabolism regulation (O_2_-site), endosperm expression (GCN4_motif and Skn-1_motif), and phloem expression (Supplementary Table S5). Taken together, these results imply that *FvYTH* genes may play a role in hormone responses, environmental stress responses, and growth and development. Additionally, the differences in the types and numbers of *cis*-elements may suggest that *FvYTH* genes may be involved in these response processes with some specificity.

### 2.6. Analysis of FvYTH Gene Expression in Different Tissues and Fruit Development Stages

**Figure 6:**
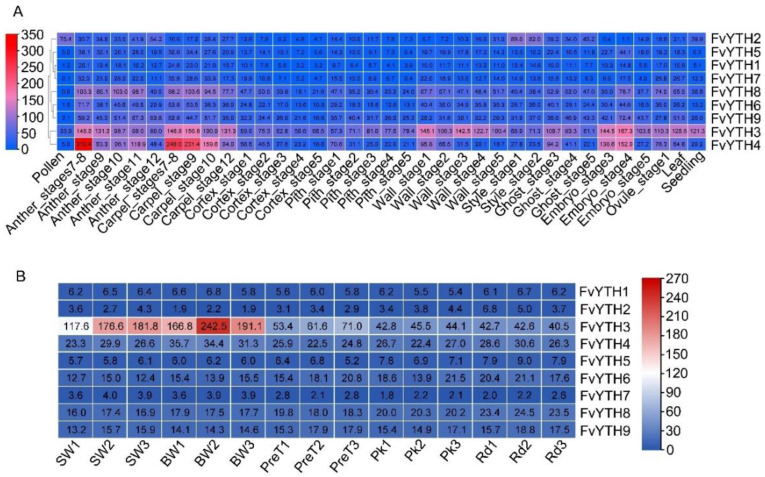
Heat map of *FvYTH* genes in strawberry. (**A**) Heat map of *FvYTH* gene expression in different tissues and organs at different development stages. The tissues and organs used for the expression profiling are indicated at the bottom of each column. (**B**) Heat map of *FvYTH* gene expression in different development stages of fruit. SW: small white stage; BW: big white stage; PreT: pre-turning stage; Pk: pink stage; Rd: red stage. The numbers 1, 2, and 3 represent three repetitions. The TPM values are shown, and the color from blue to red shows the scaled TPM values.

**Figure 7:**
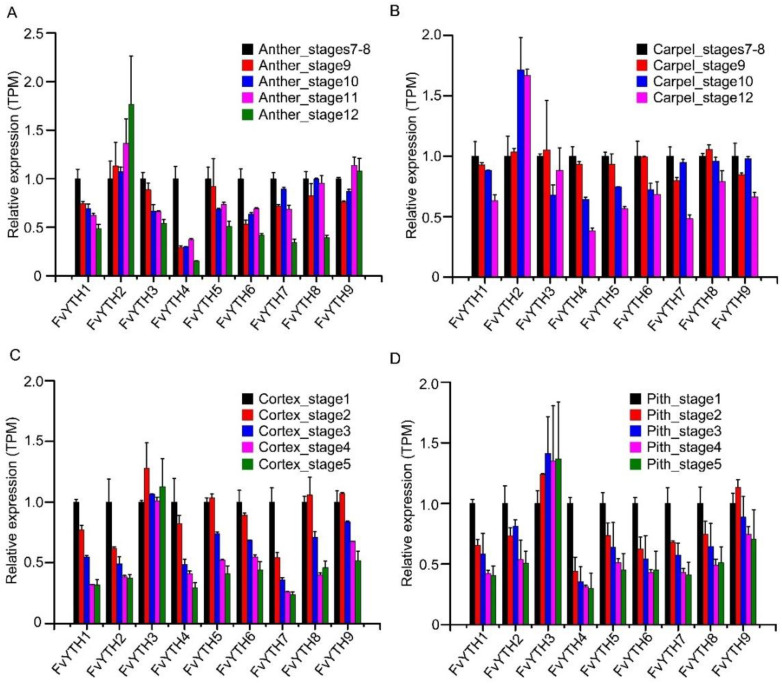
Expression trends of *FvYTH* genes in different stages of organ development. The expression trends of *FvYTH* genes in different development stages of anther (**A**), carpel (**B**), cortex (**C**), and pith (**D**). The TPM value of the selected first stage was normalized as 1.

The function of YTH proteins in plants other than *Arabidopsis* and rice has been less studied. To further investigate in which tissues or developmental processes YTH proteins may play a role in strawberry, we analyzed the expression of *FvYTH* genes with the help of transcriptome data available for forest strawberry. The analysis showed that although the expression levels of *FvYTH* genes varied, they were expressed in all the tissue sites examined. The *FvYTH3* gene was expressed at higher levels in all tissues compared to other *YTH* genes except *FvYTH4*, which was expressed at higher levels in anthers, carpels, and embryos (Figure 6A). Similarly, the *FvYTH8* gene was expressed in higher levels in anthers and carpels than in other tissues. The *FvYTH2* gene was significantly more expressed in pollen and styles than in other tissues (Figure 6A). By analyzing the expression trends of *YTH* genes during tissue development, the results showed that the expression of *FvYTH2* gradually increased during the development of carpels and anthers, while the expression of the most of remaining *YTH* genes was higher in early development than in late development (Figure 7A,B). These patterns of YTH gene expression were also observed during carpel wall development stages (Supplementary Figure S5A). The expression trend of *FvYTH2* during embryo development was gradually increased as in other tissues such as anther, carpel, and pith, but the remaining *YTH* genes were first increased and then decreased during embryo development (Supplementary Figure S5B). Among the five stages of early receptacle development, the expression of *FvYTH3* genes in the cortex and pith was relatively stable and did not change drastically. However, the expression of all other *YTH* genes decreased as the developmental process progressed (Figure 6A and Figure 7C,D). The diversity in the expression amounts and expression trends of *YTH* genes implies that their roles may vary during tissue development.

In addition, we also analyzed the expression of *YTH* genes from white fruits to the ripening stages. The results showed that the expression trends of all *YTH* genes except *FvYTH3* and *FvYTH7* were relatively stable at different stages of fruit development, while *FvYTH3* and *FvYTH7* genes gradually decreased as the fruit reached maturity (Supplementary Figure S5C). Although there was a decrease in the expression level of *FvYTH3*, the TPM value was higher than that of the remaining eight *YTH* genes at different stages of fruit development (Figure 6B). These results imply that *FvYTH3* genes may play a more critical role during fruit development and ripening compared with other genes.

## 3. Discussion

Methylation of DNA is known to be a ubiquitous epigenetic phenomenon regulating gene expression. It is increasingly found that not only DNA but also RNA methylation is a widespread epigenetic modality in plants and animals, and the effective recognition of RNA-binding proteins is often required for RNA to play a specific role after methylation [30]. The m6A modification is one of the most abundant types of the many different RNA modifications. Therefore, it is particularly important to identify YTH proteins which can bind RNA molecules with m6A modifications. Studies have found 13 YTH proteins in *Arabidopsis* [25], 12 in rice [25], 9 in tomato [31], 26 in apple [32], and up to 39 in common wheat [33]. Although there have been relevant studies on YTH in these species, *Arabidopsis* and rice are used as model plants for dicotyledons and monocotyledons, which are not suitable as model plants for studying fruits. Apple is a perennial woody plant, which requires a long time for studying fruit development. The strawberry is a pseudo-fruit, and the achenes located on the fruit surface are its biological definition of seeds, while the fruit is formed by receptacle expansion and development. The diploid forest strawberry has a small genome, has continuous flowering characteristics, and its ease of genetic manipulation makes it an ideal model plant for studying berry plants. Therefore, it is advantageous and necessary to conduct YTH studies in forest strawberry. In this study, we identified a total of nine *YTH* genes in forest strawberry, which is almost twice the number of human *YTH* genes. The presence of more YTH proteins in plants compared to mammals seems to be a general phenomenon. In humans, five YTH proteins were identified, including YTHDC1 and YTHDC2 in the DC group and YTHDF1/2/3 in the DF group [4]. Although segmental duplication was not found in our study, *FvYTH3* and *FvYTH4* were found to be generated by tandem duplication (Figure 1A), which is consistent with the similarity of their gene structure and motif composition (Figure 2 and Figure 3). Li et al. found that tandem duplication was not involved in the evolution of the *OsYTHs* gene family in rice, and both tandem duplication and segmental duplication events are involved in the evolution of the *AtYTHs* gene family in *Arabidopsis* [25]. This shows the evolutionary differences between strawberry and the model plants *Arabidopsis* and rice. The increase in the number of YTH proteins in plants may be due to the fact that *YTH* genes did not simply undergo gene duplication during evolution, but these duplications generated new genes and may have generated new functions during evolution. This would explain why they were retained.

The homology search did not find any YTHDC2 homologs in plants; only YTHDC1 homologs were found. Most dicotyledonous plants have more than two YTHDC1 homologs, e.g., *Arabidopsis* has two and tomato has five [25,31]. In contrast, monocotyledonous plants have only one to two YTHDC1 homologs, e.g., rice has only one [25]. Our results show that two DC subfamily YTH proteins, FvYTH1 and FvYTH7, are also present in strawberry, and FvYTH1 contains a conserved Znf-CCCH domain at its N-terminus compared with FvYTH7 (Figure 2A). Among the RNA-binding proteins in plants, in addition to YTH proteins, glycine-rich zinc finger-type proteins are also capable of binding RNA molecules. These zinc finger RNA-binding proteins usually contain a CCCH-type domain [30]. Given that both types of proteins may bind RNA molecules, it is necessary to explore further in future studies whether the inclusion of both CCCH-type domains and YTH domains plays a special role, such as whether they have a stronger ability to bind RNA than proteins having only a YTH domain. It has been shown that a key mechanism by which m6A-modified mRNAs are regulated is through a liquid–liquid phase separation process [34,35,36]. By studying the amino acid sequences of YTH proteins, it was found that the N termini of YTH proteins all contain a large glutamine/proline/glycine (Q/P/G)-rich low-complexity structural domain that is essential for the phase separation of YTH proteins [37]. It was shown that the presence of an inherently disordered region of the protein sequence makes the protein more susceptible to droplet formation [38]. Prediction of the intrinsically disordered region in the N-terminal sequence (the sequence before the YTH domain) of strawberry YTH proteins revealed that all YTH proteins except FvYTH7 contain this disorder region. Analysis of the amino acid composition of this region revealed that this region is enriched with six amino acids, G/N/P/Q/S/Y (Supplementary Figure S6). This implies that the recognition of m6A-modified RNA by strawberry YTH proteins may also function in a similar liquid–liquid phase separation manner.

Phylogenetic analysis showed that FvYTH3 and FvYTH4 belong to the same subfamily as ECT2/3/4 in *Arabidopsis*, which was found to regulate leaf morphology and developmental processes [26]. This leads us to recall that *FvYTH3* and *FvYTH4* are significantly more expressed in flowers, leaves, and seedlings than other *YTH* genes (Figure 6A). This may suggest that these two genes may have similar functions to ECT2/3/4. By analyzing the changes in m6A levels of mRNA, it was found that the ripening-promoting effects of MTA and MTB were mainly mediated by the m6A methylation modification of NECD5, AREB1, and ABAR, which promoted ABA synthesis and signaling [20]. Strawberry is a non-respiratory transgressive fruit, and ABA plays a critical role in its ripening process. By analyzing the promoters of *FvYTH* genes, we found that the promoter regions of all genes contain multiple hormone-response elements, which include ABA-response elements (Figure 5). This implies a necessary interconnection of YTH with ABA and fruit ripening. By analyzing the expression profile of *YTH* genes in strawberry, we found that *FvYTH3/4/6/8/9* were more highly expressed in anthers and carpels (Figure 6A). However, Li et al. found that most of the *AtYTHs* genes have high expression potentials during the senescence stage, and in rice, most *OsYTHs* genes are lowly expressed at the flowering stage [25]. These phenomena suggest that the expression of *YTH* genes in different species is distinctive. In addition, studies in *Arabidopsis*, rice, and apple have focused on the expression changes of *YTH* genes in different tissues and in response to biotic or abiotic stresses, and the analysis of *YTH* gene expression during fruit development is lacking. We analyzed the expression changes of *YTH* genes at different stages of fruit development, which provides clues to the role of *YTH* genes in fruit development. Our results showed that all *FvYTH* genes were expressed, but the expression of *FvYTH3* was higher than the other eight *YTH* genes in all stages. Moreover, the expression of *FvYTH3* showed a decreasing trend as the fruit matured (Figure 6B and Figure S5C). In fact, among all *FvYTH* genes, *FvYTH3* is unique in that its expression is higher in all tissues and in fruit development and ripening (Figure 6), and the distinctive expression level of *FvYTH3* implies that it may have multiple roles in strawberry growth and development. In other words, it may not only regulate leaf morphology and developmental processes like the homologous gene ECT2/3/4 but also play a role in regulating fruit development and ripening. In addition, although the TPM value of *FvYTH7* was not as high as that of *FvYTH3* (Figure 6B), it also showed a decreasing trend during fruit development and ripening just like *FvYTH3* (Supplementary Figure S5C). Considering that FvYTH7 belongs to the YTHDC subfamily and FvYTH3 belongs to the YTHDF subfamily, whether FvYTH7 has a role in fruit development and ripening is also a research direction worth exploring in the future.

## 4. Materials and Methods

### 4.1. Identification and Protein Properties of YTH Proteins in Strawberry

The genome of forest strawberry version 4 was obtained from the GDR database (https://www.rosaceae.org/ (accessed on 22 February 2023)). We used two mechanisms to obtain information on m6A-related genes of strawberry. First, we used the m6A reader proteins of *Arabidopsis thaliana* to blast against the protein sequences of strawberry to get homologous proteins. Second, the Pfam ID (PF04146) of the YTH conserved domain of the m6A reader protein and the HMM search function in TBtools software were used for the identification of m6A-reading genes in strawberry. Redundant sequences were removed, and the remaining putative YTH protein sequences were used as queries to search against Pfam, SMART, and InterPro databases to verify the presence of the YTH domain (PF04146). The isoelectric points, molecular weights, instability, and hydropathicity of the corresponding m6A reader proteins were analyzed with tools from ExPASY. The subcellular localizations were predicted by Plant-mPLoc (http://www.csbio.sjtu.edu.cn/bioinf/plant-multi/ (accessed on 22 February 2023)) and LOCALIZER (https://localizer.csiro.au/ (accessed on 22 February 2023)). The gene sequences of *FvYTH*s are shown in Supplementary Table S6.

### 4.2. Chromosome Localization, Synteny Relationship, Gene Structure, Conserved Domain, and Motif Analysis

The conserved domains of the YTH proteins were analyzed by Batch Web CD-Search Tool (https://www.ncbi.nlm.nih.gov/Structure/bwrpsb/bwrpsb.cgi (accessed on 22 February 2023)), and the conserved motifs were obtained from the online Multiple Expectation Maximization for Motif Elicitation (MEME) tool (http://meme-suite.org/tools/meme (accessed on 22 February 2023)). The chromosomal localization of *YTH* genes, gene structure, synteny relationships, conserved domains, and motifs were shown by using TBtools [39].

### 4.3. Phylogenetic Analysis and Cis-Acting Element Prediction in Promoters

The YTH protein sequences of rice and *Arabidopsis* were obtained from the previous literature [40]. Multiple sequence alignment of YTH proteins and phylogenetic analysis were performed with default parameters of MEGA11, and the phylogenetic tree was visualized by Evolview [41]. The 2000 bp upstream of the start codon was submitted as promoters to the PlantCare website for analysis of *cis-*acting elements [42]. The results of the analysis were visualized by TBtools [39].

### 4.4. Expression Analysis of FvYTH Gene

*FvYTH* gene expression changes in flowers, achenes, receptacle development, and leaves and seedlings were obtained from the gene expression database of strawberry (https://bar.utoronto.ca/efp_strawberry/cgi-bin/efpWeb.cgi (accessed on 22 February 2023)). The expression levels of *FvYTH* genes from white fruits to the ripening stage were obtained by analyzing previously published RNA-Seq data [43]. The accession number of raw RNA-Seq data is PRJNA522346. Transcripts per million (TPM) values were visualized using the heatmap function of TBtools [39].

## 5. Conclusions

Nine YTH proteins are present in strawberries. Seven of them belong to the YTHDF subfamily, and these seven YTH proteins contain only one YTH structural domain located at the C-terminus, with no other typical structural domains present. The other two YTHs, FvYTH1 and FvYTH7, belong to the YTHDC subfamily, and the YTH domains of both proteins are located in the middle of their protein sequences. However, unlike the YTHDF subfamily, the FvYTH1 protein also has a Znf-CCCH domain. Gene expression analysis showed that *FvYTH* genes were widely expressed in the examined tissues and during their development. The *FvYTH3* gene was expressed at a high level in almost all tissues, while *FvYTH4*, which belongs to a tandem repeat gene pair with *FvYTH3*, was expressed at a lower level during fruit development but at a similar level to *FvYTH3* in other tissues.

## Figures and Tables

**Table 1:** Protein properties of YTH proteins in strawberry.

Gene Name	Gene ID	Molecular Weigh (Da)	Isoelectric Point	Length (aa)
FvYTH1	FvH4_3g09980	75,927.17	6.37	689
FvYTH2	FvH4_3g13840	65,995.31	6.37	592
FvYTH3	FvH4_3g45840	84,983.28	8.53	773
FvYTH4	FvH4_3g45841	69,024.37	5.67	631
FvYTH5	FvH4_4g00190	75,181.65	5.12	682
FvYTH6	FvH4_4g21030	75,433.06	6.1	686
FvYTH7	FvH4_5g01140	40,460.09	6.24	355
FvYTH8	FvH4_5g30420	58,841.15	6.44	534
FvYTH9	FvH4_6g36470	72,549.24	5.51	659

## Data Availability

The data presented in this study are available in the graphs and tables provided in the manuscript.

## Supplementary Materials

The following supporting information can be downloaded at: https://www.mdpi.com/article/10.3390/plants12071449/s1, Figure S1: Showing the logos of ten motifs in FvYTH proteins. Figure S2: Phylogenetic analysis of YTHs in strawberry and humans. Figure S3: Sequence alignments YTHDFs in strawberry and humans. Figure S4: Sequence alignments YTHDCs in strawberry and humans. Figure S5: The expression trends of FvYTH genes in different development stages. Figure S6: The bias in composition of the N terminal of FvYTH proteins compared with all strawberry proteins. Table S1: The identification of FvYTH genes in strawberry. Table S2: The properties of YTH proteins. Table S3: The synteny relationships between forest strawberry and Arabidopsis. Table S4: The sequences of YTH domain from strawberry, rice, Arabidopsis. Table S5: The identification of cis-elements in the promoters of FvYTH genes. Table S6: The CDS sequences of FvYTH genes.

## References

[1] Bannister A.J., Kouzarides T. (2011). Regulation of Chromatin by Histone Modifications. Cell Res..

[2] Goldberg A.D., Allis C.D., Bernstein E. (2007). Epigenetics: A Landscape Takes Shape. Cell.

[3] Boccaletto P., Machnicka M.A., Purta E., Piątkowski P., Bagiński B., Wirecki T.K., de Crécy-Lagard V., Ross R., Limbach P.A., Kotter A. (2018). MODOMICS: A Database of RNA Modification Pathways. 2017 Update. Nucleic Acids Res..

[4] Fu Y., Dominissini D., Rechavi G., He C. (2014). Gene Expression Regulation Mediated through Reversible M6A RNA Methylation. Nat. Rev. Genet..

[5] Bokar J.A., Shambaugh M.E., Polayes D., Matera A.G., Rottman F.M. (1997). Purification and CDNA Cloning of the AdoMet-Binding Subunit of the Human MRNA (N6-Adenosine)-Methyltransferase. RNA.

[6] Bujnicki J.M., Feder M., Radlinska M., Blumenthal R.M. (2002). Structure Prediction and Phylogenetic Analysis of a Functionally Diverse Family of Proteins Homologous to the MT-A70 Subunit of the Human MRNA:M(6)A Methyltransferase. J. Mol. Evol..

[7] Liu J., Yue Y., Han D., Wang X., Fu Y., Zhang L., Jia G., Yu M., Lu Z., Deng X. (2014). A METTL3-METTL14 Complex Mediates Mammalian Nuclear RNA N6-Adenosine Methylation. Nat. Chem. Biol..

[8] Ping X.-L., Sun B.-F., Wang L., Xiao W., Yang X., Wang W.-J., Adhikari S., Shi Y., Lv Y., Chen Y.-S. (2014). Mammalian WTAP Is a Regulatory Subunit of the RNA N6-Methyladenosine Methyltransferase. Cell Res..

[9] Zhang Z., Mei Y., Hou M. (2022). Knockdown RBM15 Inhibits Colorectal Cancer Cell Proliferation and Metastasis Via N6-Methyladenosine (M6A) Modification of MyD88 MRNA. Cancer Biother. Radio..

[10] Mauer J., Luo X., Blanjoie A., Jiao X., Grozhik A.V., Patil D.P., Linder B., Pickering B.F., Vasseur J.-J., Chen Q. (2017). Reversible Methylation of M6Am in the 5′ Cap Controls MRNA Stability. Nature.

[11] Jia G., Fu Y., Zhao X., Dai Q., Zheng G., Yang Y., Yi C., Lindahl T., Pan T., Yang Y.-G. (2011). N6-Methyladenosine in Nuclear RNA Is a Major Substrate of the Obesity-Associated FTO. Nat. Chem. Biol..

[12] Jia G., Fu Y., He C. (2013). Reversible RNA Adenosine Methylation in Biological Regulation. Trends Genet..

[13] Patil D.P., Pickering B.F., Jaffrey S.R. (2018). Reading M6A in the Transcriptome: M6A-Binding Proteins. Trends Cell Biol..

[14] Lin Z., Hsu P.J., Xing X., Fang J., Lu Z., Zou Q., Zhang K.-J., Zhang X., Zhou Y., Zhang T. (2017). Mettl3-/Mettl14-Mediated MRNA N6-Methyladenosine Modulates Murine Spermatogenesis. Cell Res..

[15] Wang X., Wu R., Liu Y., Zhao Y., Bi Z., Yao Y., Liu Q., Shi H., Wang F., Wang Y. (2020). M ^6^ A MRNA Methylation Controls Autophagy and Adipogenesis by Targeting *Atg5* and *Atg7*. Autophagy.

[16] Mu H., Zhang T., Yang Y., Zhang D., Gao J., Li J., Yue L., Gao D., Shi B., Han Y. (2021). METTL3-Mediated MRNA N6-Methyladenosine Is Required for Oocyte and Follicle Development in Mice. Cell Death Dis..

[17] Han Z., Wang X., Xu Z., Cao Y., Gong R., Yu Y., Yu Y., Guo X., Liu S., Yu M. (2021). ALKBH5 Regulates Cardiomyocyte Proliferation and Heart Regeneration by Demethylating the MRNA of YTHDF1. Theranostics.

[18] Shen L., Liang Z., Gu X., Chen Y., Teo Z.W.N., Hou X., Cai W.M., Dedon P.C., Liu L., Yu H. (2016). N6-Methyladenosine RNA Modification Regulates Shoot Stem Cell Fate in Arabidopsis. Dev. Cell.

[19] Duan H.-C., Wei L.-H., Zhang C., Wang Y., Chen L., Lu Z., Chen P.R., He C., Jia G. (2017). ALKBH10B Is an RNA *N* ^6^ -Methyladenosine Demethylase Affecting Arabidopsis Floral Transition. Plant Cell.

[20] Zhou L., Tang R., Li X., Tian S., Li B., Qin G. (2021). N6-Methyladenosine RNA Modification Regulates Strawberry Fruit Ripening in an ABA-Dependent Manner. Genome Biol..

[21] Hou N., Li C., He J., Liu Y., Yu S., Malnoy M., Mobeen Tahir M., Xu L., Ma F., Guan Q. (2022). MdMTA-mediated m ^6^ A Modification Enhances Drought Tolerance by Promoting MRNA Stability and Translation Efficiency of Genes Involved in Lignin Deposition and Oxidative Stress. New Phytol..

[22] Zhou L., Tian S., Qin G. (2019). RNA Methylomes Reveal the M6A-Mediated Regulation of DNA Demethylase Gene SlDML2 in Tomato Fruit Ripening. Genome Biol..

[23] Liao S., Sun H., Xu C. (2018). YTH Domain: A Family of N 6 -Methyladenosine (m 6 A) Readers. Genom. Proteom. Bioinf..

[24] Meyer K.D., Jaffrey S.R. (2017). Rethinking m ^6^ A Readers, Writers, and Erasers. Annu. Rev. Cell Dev. Biol..

[25] Li D., Zhang H., Hong Y., Huang L., Li X., Zhang Y., Ouyang Z., Song F. (2014). Genome-Wide Identification, Biochemical Characterization, and Expression Analyses of the YTH Domain-Containing RNA-Binding Protein Family in Arabidopsis and Rice. Plant Mol. Biol. Rep..

[26] Arribas-Hernández L., Bressendorff S., Hansen M.H., Poulsen C., Erdmann S., Brodersen P. (2018). An m^6^A-YTH Module Controls Developmental Timing and Morphogenesis in Arabidopsis. Plant Cell.

[27] Hou Y., Sun J., Wu B., Gao Y., Nie H., Nie Z., Quan S., Wang Y., Cao X., Li S. (2021). CPSF30-L-Mediated Recognition of MRNA M6A Modification Controls Alternative Polyadenylation of Nitrate Signaling-Related Gene Transcripts in Arabidopsis. Mol. Plant.

[28] Song P., Yang J., Wang C., Lu Q., Shi L., Tayier S., Jia G. (2021). Arabidopsis N6-Methyladenosine Reader CPSF30-L Recognizes FUE Signals to Control Polyadenylation Site Choice in Liquid-like Nuclear Bodies. Mol. Plant.

[29] Guo T., Liu C., Meng F., Hu L., Fu X., Yang Z., Wang N., Jiang Q., Zhang X., Ma F. (2022). The m ^6^ A Reader MhYTP2 Regulates *MdMLO19* MRNA Stability and Antioxidant Genes Translation Efficiency Conferring Powdery Mildew Resistance in Apple. Plant Biotechnol. J..

[30] Muthusamy M., Kim J.-H., Kim J.A., Lee S.-I. (2021). Plant RNA Binding Proteins as Critical Modulators in Drought, High Salinity, Heat, and Cold Stress Responses: An Updated Overview. Int. J. Mol. Sci..

[31] Yin S., Ao Q., Tan C., Yang Y. (2021). Genome-Wide Identification and Characterization of YTH Domain-Containing Genes, Encoding the M6A Readers, and Their Expression in Tomato. Plant Cell Rep..

[32] Wang N., Yue Z., Liang D., Ma F. (2014). Genome-Wide Identification of Members in the YTH Domain-Containing RNA-Binding Protein Family in Apple and Expression Analysis of Their Responsiveness to Senescence and Abiotic Stresses. Gene.

[33] Sun J., Bie X.M., Wang N., Zhang X.S., Gao X.-Q. (2020). Genome-Wide Identification and Expression Analysis of YTH Domain-Containing RNA-Binding Protein Family in Common Wheat. BMC Plant Biol..

[34] Fu Y., Zhuang X. (2020). M6A-Binding YTHDF Proteins Promote Stress Granule Formation. Nat. Chem. Biol..

[35] Wang J., Wang L., Diao J., Shi Y.G., Shi Y., Ma H., Shen H. (2020). Binding to M6A RNA Promotes YTHDF2-Mediated Phase Separation. Protein Cell.

[36] Gao Y., Pei G., Li D., Li R., Shao Y., Zhang Q.C., Li P. (2019). Multivalent M6A Motifs Promote Phase Separation of YTHDF Proteins. Cell Res..

[37] Zaccara S., Ries R.J., Jaffrey S.R. (2019). Reading, Writing and Erasing MRNA Methylation. Nat. Rev. Mol. Cell Bio..

[38] Boeynaems S., Alberti S., Fawzi N.L., Mittag T., Polymenidou M., Rousseau F., Schymkowitz J., Shorter J., Wolozin B., Van Den Bosch L. (2018). Protein Phase Separation: A New Phase in Cell Biology. Trends Cell Biol..

[39] Chen C., Chen H., Zhang Y., Thomas H.R., Frank M.H., He Y., Xia R. (2020). TBtools: An Integrative Toolkit Developed for Interactive Analyses of Big Biological Data. Mol. Plant.

[40] Yue H., Nie X., Yan Z., Weining S. (2019). N6-Methyladenosine Regulatory Machinery in Plants: Composition, Function and Evolution. Plant Biotechnol. J..

[41] Subramanian B., Gao S., Lercher M.J., Hu S., Chen W.-H. (2019). Evolview v3: A Webserver for Visualization, Annotation, and Management of Phylogenetic Trees. Nucleic. Acids Res..

[42] Lescot M., Déhais P., Thijs G., Marchal K., Moreau Y., Van de Peer Y., Rouzé P., Rombauts S. (2002). PlantCARE, a Database of Plant *Cis-*Acting Regulatory Elements and a Portal to Tools for in Silico Analysis of Promoter Sequences. Nucleic. Acids Res..

[43] Gu T., Jia S., Huang X., Wang L., Fu W., Huo G., Gan L., Ding J., Li Y. (2019). Transcriptome and Hormone Analyses Provide Insights into Hormonal Regulation in Strawberry Ripening. Planta.

