# Supplementary material for: Comprehensive Identification and Expression Analysis of the YTH Family of RNA-Binding Proteins in Strawberry
Source: Plants (Basel). 2023 Mar 25;12(7):1449. doi: 10.3390/plants12071449 (PMC10097400; doi:10.3390/plants12071449)
Supplement: Supplementary file 1 [file plants-12-01449-s001.zip › plants-2267835-supplementary.pdf]

Supplementary Informations

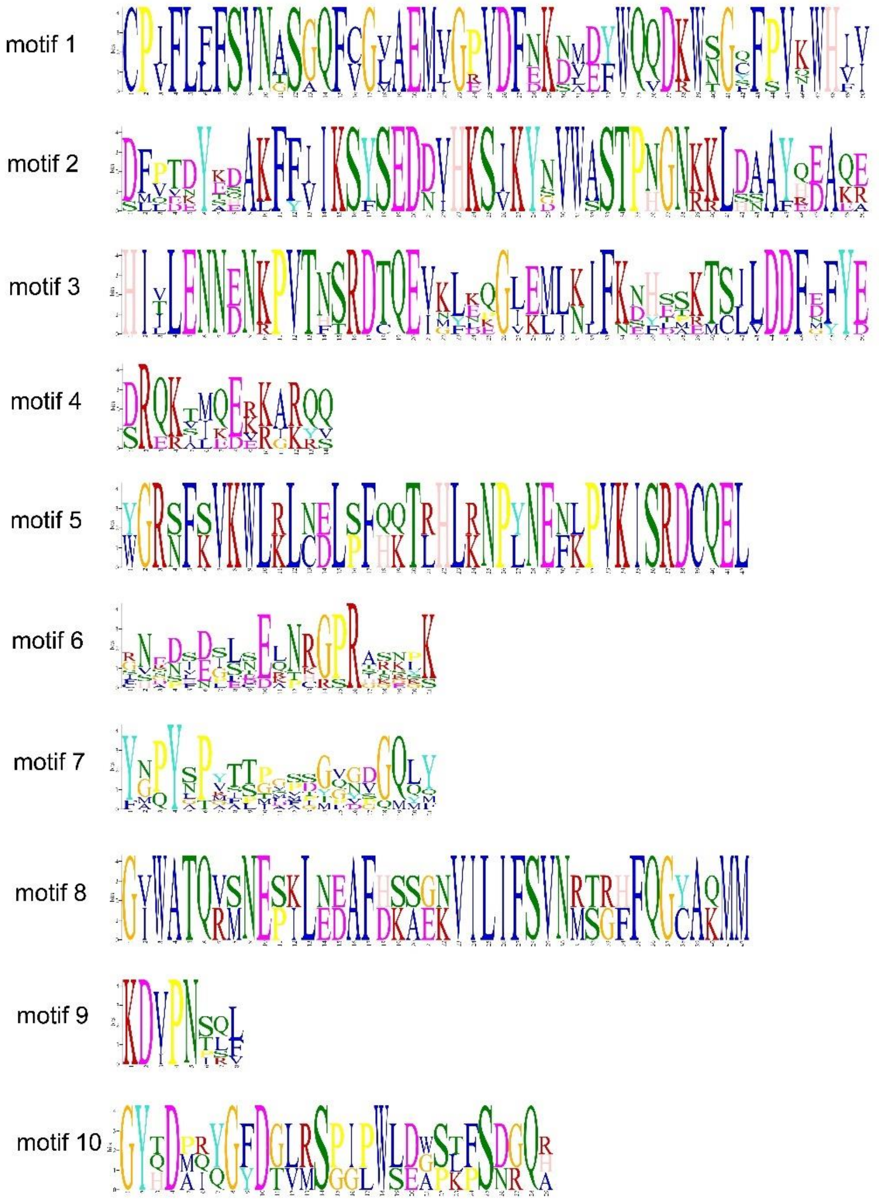

Supplementary Figure S1. Showing the logos of ten motifs in FvYTH proteins. The X axis of sequence logos refers to the amino acid with the highest frequency and the Y axis represents the relative frequency of the corresponding amino acid.

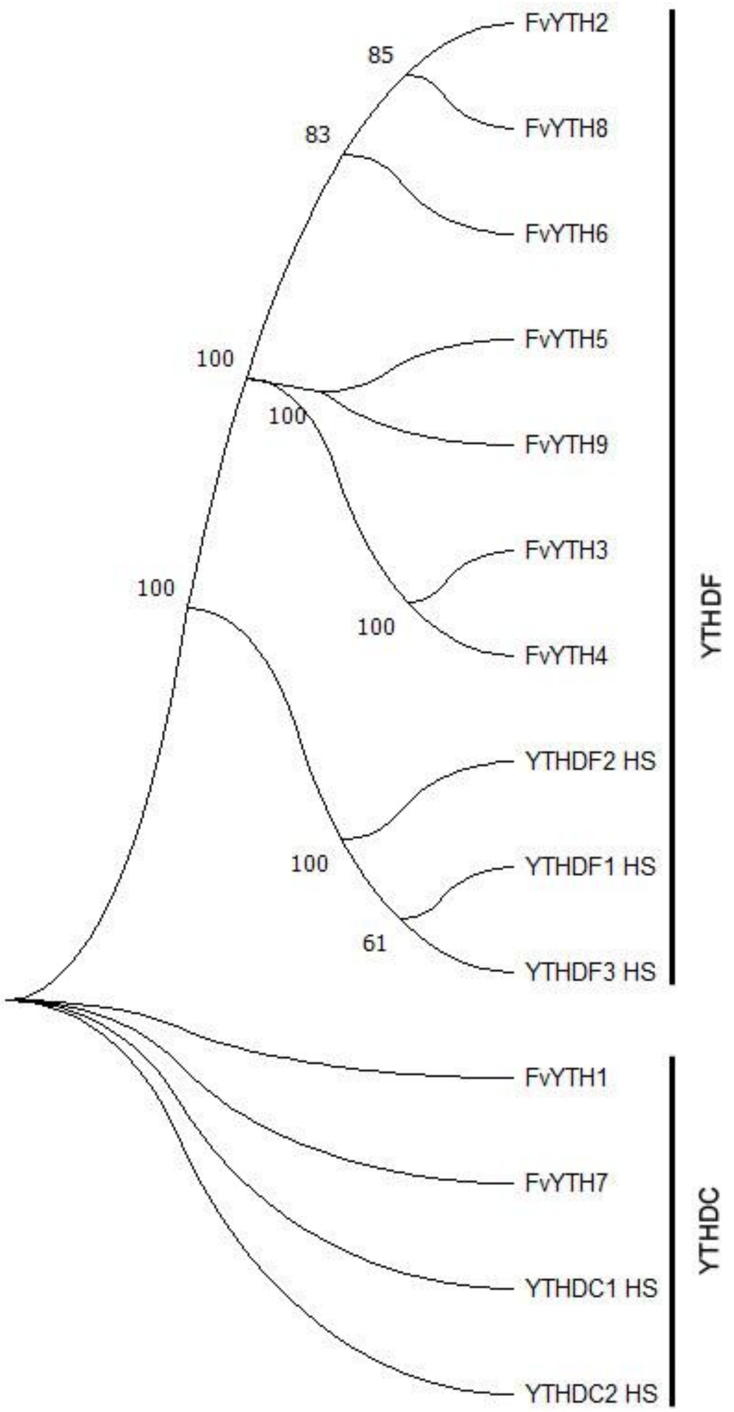

Supplementary Figure S2. Phylogenetic analysis of YTHs in strawberry and humans. The sequences of YTH proteins from strawberry and humans were used to constructed phylogenetic tree by NJ method, with 1000 bootstrap replications. HS represents humans.

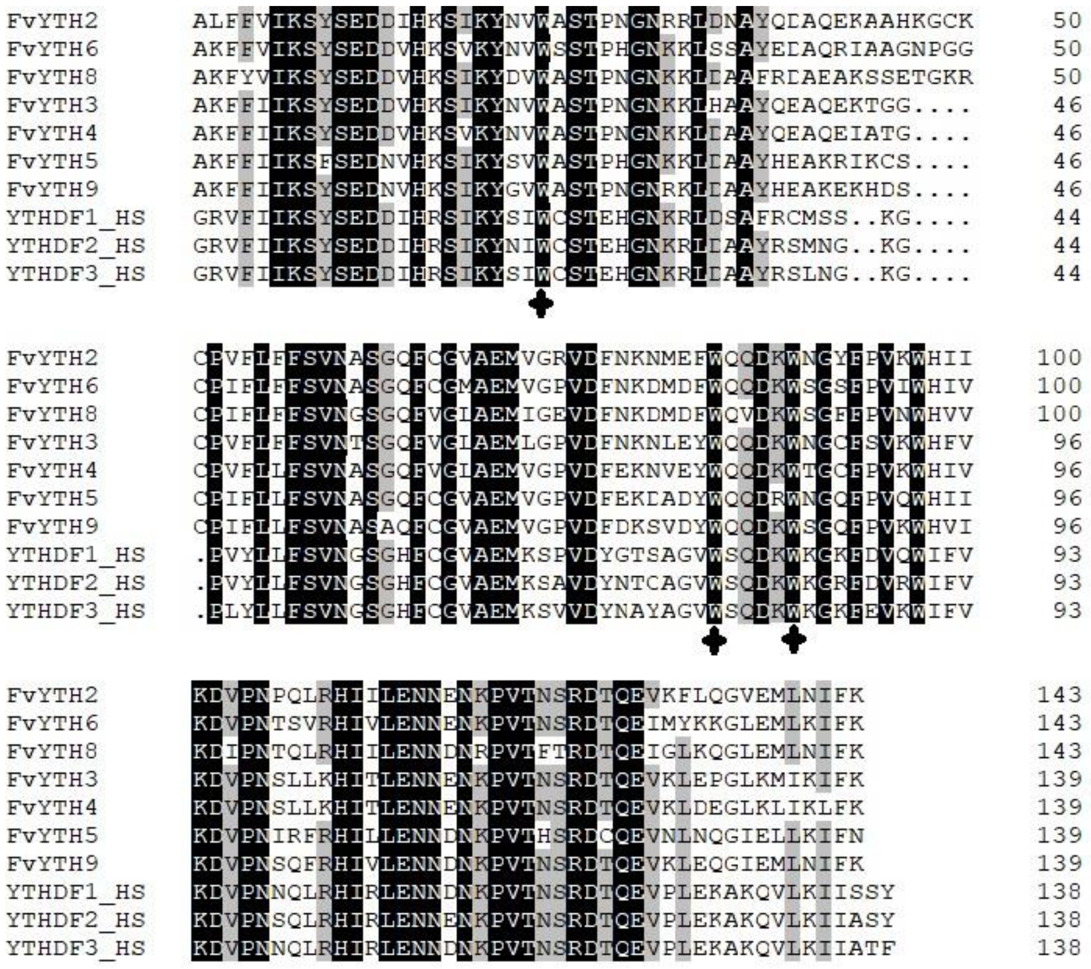

Supplementary Figure S3. Sequence alignments YTHDFs in strawberry and humans. The conserved amino acids involved in cage formation are indicated with asterisks.

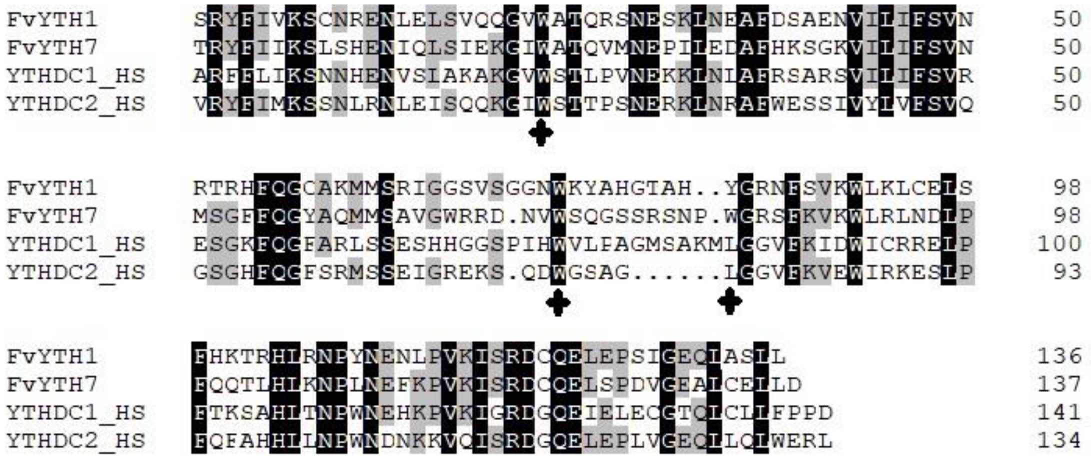

Supplementary Figure S4. Sequence alignments YTHDCs in strawberry and humans. The conserved amino acids involved in cage formation are indicated with asterisks.

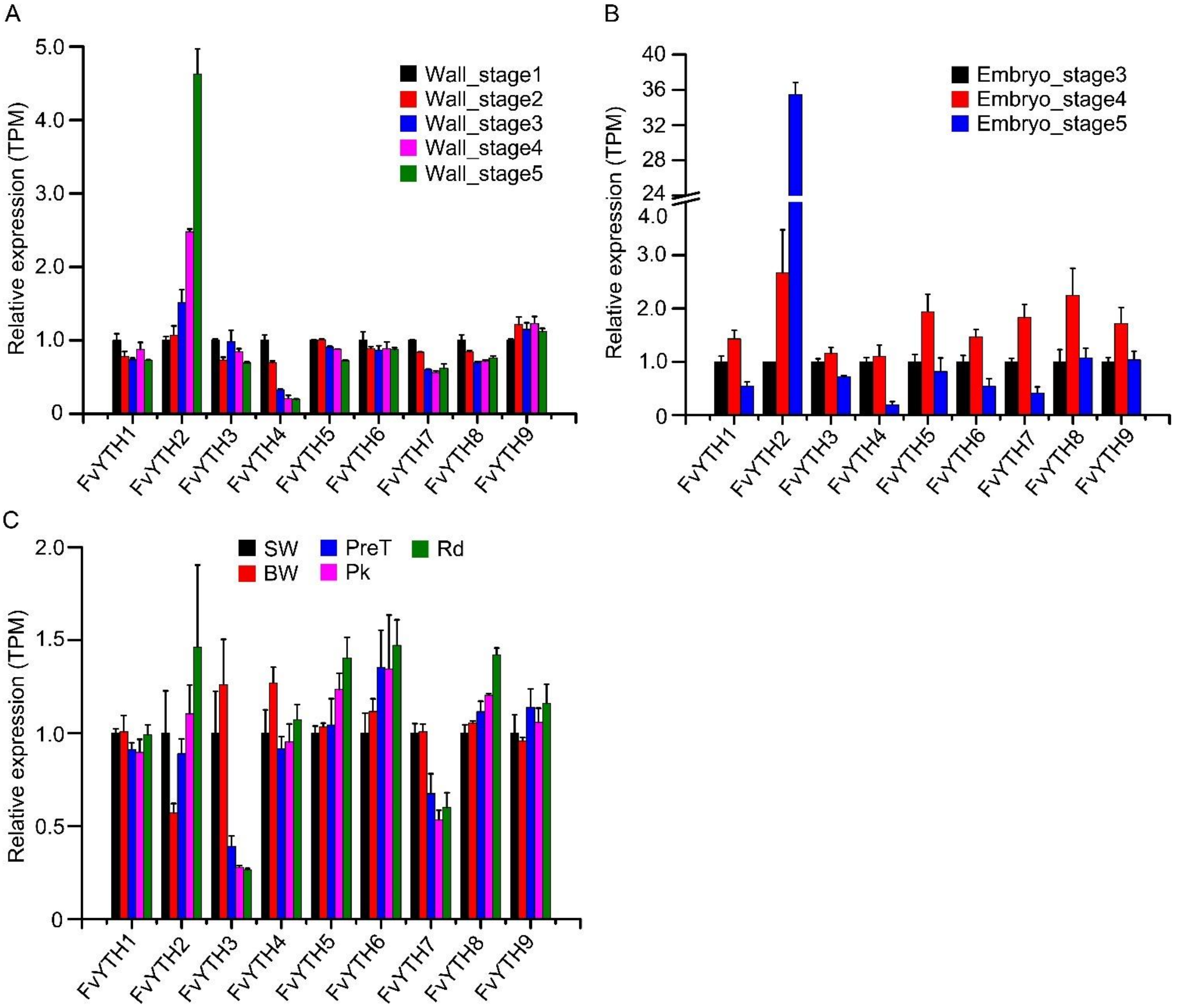

Supplementary Figure S5. The expression trends of *FvYTH* genes in different development stages. The expression trends of *FvYTH* genes in different development stages of carpel wall (A), embryo (B), and fruit (C). The TPM value of the selected first stage was

normalized as 1. SW: small white stage; BW: big white stage; PreT: pre-turning stage; Pk: pink stage; Rd: red stage.

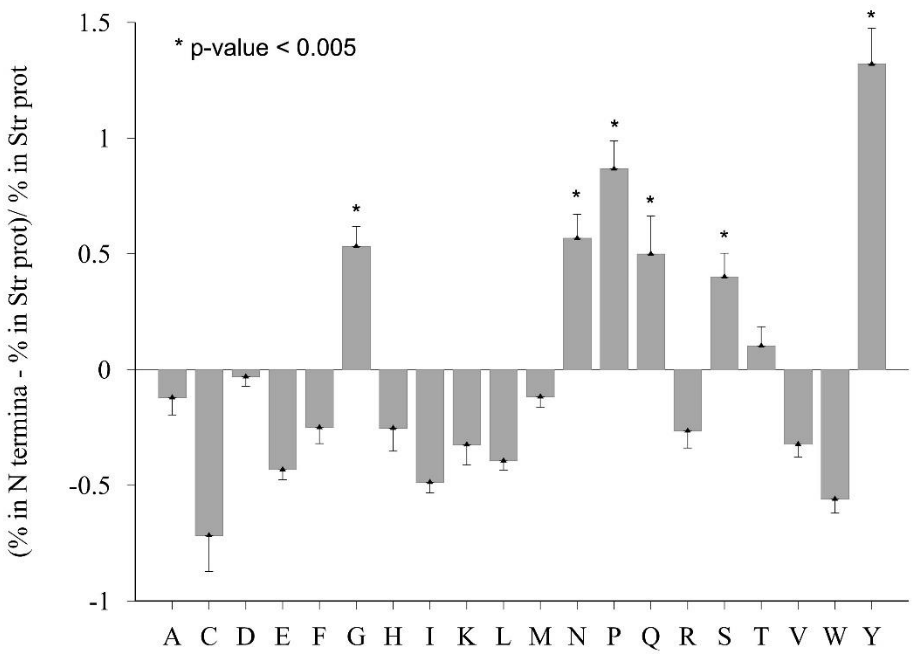

Supplementary Figure S6. The bias in composition of the N terminal of YTH proteins compared with all strawberry proteins. The y axis represents the proportion of each amino acid in the N-terminal region compared with a data base of all Strawberry proteins. The richer amino acids are statistically ($P<0.005$) overrepresented in this region. Statistical significance associated with a specific enrichment or depletion is estimated using the two-sample t test including a Bonferroni correction according to the Composition Profiler website (http://www.cprofiler.org (accessed on 22/2/2023)).

Supplementary Table S1. The identification of *FvYTH* genes in strawberry.

Target	Query Name	Accession	E-value	score
FvH4_3g09980	YTH	PF04146.15	5.10E-47	161.7
FvH4_3g13840	YTH	PF04146.15	4.70E-39	135.8
FvH4_3g45840	YTH	PF04146.15	1.90E-39	137.1
FvH4_3g45841	YTH	PF04146.15	8.40E-41	141.5
FvH4_4g00190	YTH	PF04146.15	2.50E-40	140
FvH4_4g21030	YTH	PF04146.15	9.60E-40	138
FvH4_5g01140	YTH	PF04146.15	8.90E-50	170.7
FvH4_5g30420	YTH	PF04146.15	2.80E-37	130
FvH4_6g36470	YTH	PF04146.15	4.20E-40	139.2

Supplementary Table S2. The properties of YTH proteins.

Gene Name	Gene ID	LOCALIZER	Plant-mPLoc	Instability Index	Grand Average of Hydropathicity（GRAVY)
FvYTH1	FvH4_3g09980	Y (RRSRHGEGKKKRRDS)	Nucleus	56.69	-0.964
FvYTH2	FvH4_3g13840	Y (KRIR)	Nucleus	40.8	-0.733
FvYTH3	FvH4_3g45840	-	Nucleus	34.12	-0.741
FvYTH4	FvH4_3g45841	Y (AKRQIRKKK)	Nucleus	35.51	-0.732
FvYTH5	FvH4_4g00190	Y (KKLDAAYHEAKRIKC)	Nucleus	49.96	-0.672
FvYTH6	FvH4_4g21030	-	Nucleus	32.59	-0.593
FvYTH7	FvH4_5g01140	Y (FKVK)	Nucleus	59.27	-0.853
FvYTH8	FvH4_5g30420	-	Nucleus	31.99	-0.696
FvYTH9	FvH4_6g36470	-	Nucleus	41.52	-0.685

Supplementary Table S3. The synteny relationships between forest strawberry and *Arabidopsis*.

chromosome	geneid		chromosome	gene name
At--Chr1	AT1G55500	==	Str--Fvb3	FvYTH3
At--Chr1	AT1G30460	==	Str--Fvb3	FvYTH1
At--Chr1	AT1G79270	==	Str--Fvb3	FvYTH2
At--Chr1	AT1G48110	==	Str--Fvb4	FvYTH6
At--Chr3	AT3G13460	==	Str--Fvb3	FvYTH3
At--Chr3	AT3G17330	==	Str--Fvb4	FvYTH6
At--Chr3	AT3G13060	==	Str--Fvb6	FvYTH9
At--Chr4	AT4G11970	==	Str--Fvb5	FvYTH7

Supplementary Table S4. The sequences of YTH domain from strawberry, rice, *Arabidopsis.*

Name	Sequences
AtECT1	AKFFVIKSYSEDDVHNCIKYGAWSSTPTGNKKLNAAYYEAKENSQECPVYLLFSVNASGQFVGLAEMVGPVDFNKTMEYWQQDKWIGCFPVKWHIIKDIPNSLLRHITLANNENKPVTNSRDTQEVNLEHGTKIIKIFK
AtECT2	AMFFIIKSYSEDDVHKSIKYNVWASTPNGNKKLAAAYQEAQQKAGGCPIFLFFSVNASGQFVGLAEMTGPVDFNTNVEYWQQDKWTGSFPLKWHIVKDVPNSLLKHITLENNENKPVTNSRDTQEVKLEQGLKIVKIFK
AtECT3	AKFYVIKSYSEDDIHKSIKYSVWSSTPNGNKKLDASYNEAKQKSDGCPVFLLFSVNTSGQFVGLAEMVGPVDFNKTVEYWQQDKWIGCFPVKWHFVKDIPNSSLRHITLENNENKPVTNSRDTQEVKLEQGIKVIKIFK
AtECT4	AKFFIIKSYSEDDVHKSIKYNVWASTPNGNKKLDAAYQEAQQKSSGCPVFLFFSVNASGQFIGLAEMKGPVDFNKNIEYWQQDKWTGSFPLKWHILKDVPNSLLKHITLEYNENKPVTNSRDTQEVKLEQGLKVVKIFK
AtECT5	AKLFIIKSYSEDNVHKSIKYNVWASTPNGNKKLDAAYREAKDEKEPCPLFLLFSVNASSQFCGVAEMVGPVDFEKSVDYWQQDKWSGQFPVKWHIIKDVPNSQFRHIILENNDNKPVTNSRDTQEVKLEQGIEMLKIFK
AtECT6	ARFFVIKSYSEDDVHKSIKYGVWSSTLNGNKKLQSVYEDAQRIATEKSRECPIFLFFSVNSSGLFCGVAEMTGPVSFDRDMDFWQQDKWSGSFPVKWHIIKDVPNSYFRHIILHNNENKPVTNSRDTQEIILKQGLEVLKLFK
AtECT7	AKFFVIKSYSEDDVHKSIKYNVWSSTLHGNKKLQSAYEDAQRIATEKSCECPIFLFFSVNASGLFCGMAEMTGPVSFDKDMDFWQQDKWSGSFPVKWHIIKDVPNSYFRHIILQNNENKPVTNSRDTQEIMLKQGLEVLKIFK
AtECT8	AIFFVIKSYSEDDIHKSIKYNVWSSTLNGNKKLDSAYQESQKKAADKSGKCPVFLFFSVNASGQFCGVAEMIGRVDYEKSMEFWQQDKWTGYFPVKWHIIKDVPNPQLRHIILENNENKPVTNSRDTQEVRLPQGNEVLNIFK
AtECT9	AKFFVIKSYSEDNVHKSIKHCVWASTKNGNKKLDAAYREAKKKDVACPVFLLFSVNASSQFCGVAEMVGPVDFNTSVEYWQQDRWSGHFPVQWLIVKDVPNSLFRHIIIESNDNKPVTNSRDTQEVGLEKGIEMLDIFISCEMRSSILDDFNFYEERQIAIQDRKARQRAV
AtECT10	AKFFIVKSFSEDNVHRSIKYNVWASTPHGNKKLDTAYRDAEKMGGKCPIFLFFSVNASGQFCGVSEMVGPVDFEKDAGYWQQDRWSGQFPVKWHIVKDIPNNRFCHILLQNNDNKPVTHSRDSQEVKLRQGIEMLRIFK
AtECT11	AKFFVIKSYSEDDVHKSIKYSVWSSTINGNKKLDAAFRDAETKTLEDGKKRPIFLFFSVNASRQFVGLAEMVGYVDFNKDLDFWQVDKWSGFFPVEWHVVKDIPNWELRHIILDNNEDKPVTHTRDTHEIKLKEGLQMLSIFK
AtECT12	TRYFIIKSLNYDNIQVSVEKGIWATQVMNEPILEGAFHKSGRVILIFSVNMSGFFQGYAEMLSPVGWRRDQIWSQGGGKNNPWGRSFKVKWLRLSELPFQKTLHLKNPLNDYKPVKISRDCQELPEDIGEALCELLD
AtCPSF30	RYFVVKSNNRENFELSVQQGVWATQRSNEAKLNEAFDSVENVILIFSVNRTRHFQGCAKMTSRIGGYIGGGNWKHEHGTAQYGRNFSVKWLKLCELSFHKTRNLRNPYNENLPVKISRDCQELEPSVGEQLAS LL
OsECT1	ARFFIIKSYSEDNVHKSIKYGVWASTTNGNKKLDSAYREAKEKEEHCPIFLLFSVNASAQFCGVAEMIGPVDFEKSVDYWQQDKWTGQFPVKWHIVKDVPNNLFRHIILENNDNKPVTNSRDTQEVKLEQGMEMLKIFK
OsECT2	RYFIVKSCNRENLEISVQQGIWATQRSNEAKLNEAFESIENVILIFSINRTRNFQGCAKMTSRIGGYIGGGNWKSAHGTAHYGRNFSIQWLKLCELSFQKTHHLRNPYNDNLPVKISRDCQELEPFIGEQLASLL
OsECT3	AKFFVIKSYTEDHVHRSIKYNVWASTASGNRKLDSAYRLAKEKEDYCPIFLFFSVNGSGQFCGVAEMIGPVDFDKSVDYWQQDK

	WSGQFPVKWHIIKDVPNNLLRHIILENNDNKPVTNSRDTQEVKLEHGLQMLTIFK
OsECT4	AKFFVIKSYTEDHVHRSIKYNVWASTASGNRKLDSAYRLAKEKEDYCPIFLFFSVNGSGQFCGVAEMIGPVDFDKSVDYWQQDK<br>WSGQFPVKWHIIKDVPNNLLRHIILENNDNKPVTNSRDTQEVKLEHGLQMLTIFK
OsECT5	AKFFMIKSYSEDDIHKGIKYNVWASTPHGNNKLDAAFREAQILIKEKGKKCPVFLFFSVNSSGQFVGLAEILGPVDFKKTMDFWKL<br>DRWNGFFPVTWHIIKDIPNRLFKHITLENNDNRIVTFSRDTQEIGLLQGLKMLKIFK
OsECT6	AKFFMIKSYSEDDVHKGIKYNVWASTPNGNNKLDAAFHEAQILMKEQGKRCPIFLFFSVNTSGQFVGLAEMLGPVDFKKTMDFW<br>QQDKWNGFFPVMWHIIKDIPNRFFKHITLENNEGKVVTFSRDTQEIGLPQGLEMLKIFK<br>VMWHIIKDIPNRFFKHITLENNEGKVVTFSRDTQEIGLPQGLEMLKIFK
OsECT7	AKFFIIKSYSEDDVHKSIKYNVWASTSNGNKKLDAAYQEAKEKSSDSSVFLLFSVNASGQFVGLAEMVGRVDFNKTLEHWQQDK<br>WTGCFPVKWHIVKDVPNSLLKHIILENNENKPVTNCRDTHEVKLEPGLQVLKIFK
OsECT8	AKFFVIKSYSEDDVHKSIKYNVWSSTPNGNKRLDAAYSDVQGRAVGKCPIFLFFSVNASGQFCGVAEMVGPVDFHKDMDFWQQ<br>DKWSGSFPVKWHLVKDVPNSTFRHIILENNENKPVTNSRDTQEIPFKSGTNMLKLFK
OsECT9	SNDLRVDYPFAKFFVIKSIGEDDVHKSIKYGVWSSSSSGNSKLDIAFKDANRIAKRNSTKCPVFLFFSVNGSGLFCGMAEMVGPV<br>DFHKDMDFWCQDKWTGSFPNNENKPVTHSRDTQEIPYVPGISMLKILK
OsECT10	AKFFVIKSYSEDDIHKSIKYNVWASTTNGNKKLDAAYQEAQAKSSKCPIFLFFSVNTSGQFVGVAEMTGAVDFEKTLEYWQQDK<br>WNGSLSLKWHIVKDVPNNILKHIILENNENKPVTNSRDTQEVNLDQGIQMLKIFK
OsECT11	ALFFVIKSYSEDDIHKSIKYNVWASTPNGNKRLDNAFKLAQERVAEKGTKCPMFLFFSVNASGQFCGVAEMVGPVDFNRNMNF<br>WQQDKWNGFFPVKWHIIKDVPNPQFRHIILENNENKPVTNSRDTQEVKFPQGSEMLNIFK
OsECT12	AKFFVIKSYSEDDVHKSIKYNVWASTPNGNKKLDAGYREAQEKSSECPVFLFFSVNTSGQFVGVAEMVGPVDFEKTVDYWQQ<br>DKWNGCFPIKWHVVKDVPNNILKHITLDNNDNKPVTNSRDTQEVKLEQGLEMLKIFK
OsECT13	ARFFIIKSYSEDNVHKSIKYGVWASTTNGNKKLDSAYREAKEKEEHCPIFLLFSVNASAQFCGVAEMIGPVDFEKSVDYWQQDKW<br>TGQFPVKWHIVKDVPNNLFRHIILENNDNKPVTNSRDTQEVKLEQGMEMLKIFK
FvYTH1	SRYFIVKSCNRENLELSVQQGVWATQRSNESKLNEAFDSAENVILIFSVNRTRHFQGCAKMMSRIGGSVSGGNWKYAHGTAHY<br>GRNFSVKWLKLCELSFHKTRHLRNPYNENLPVKISRDCQELEPSIGEQLASLL
FvYTH2	ALFFVIKSYSEDDIHKSIKYNVWASTPNGNRRLDNAYQDAQEKAAHKGCKCPVFLFFSVNASGQFCGVAEMVGRVDFNKNMEF<br>WQQDKWNGYFPVKWHIIKDVPNPQLRHIILENNENKPVTNSRDTQEVKFLQGVEMLNIFK
FvYTH3	AKFFIIKSYSEDDVHKSIKYNVWASTPNGNKKLHAAYQEAQEKTGGCPVFLFFSVNTSGQFVGLAEMLGPVDFNKNLEYWQQD<br>KWNGCFSVKWHFVKDVPNSLLKHITLENNENKPVTNSRDTQEVKLEPGLKMIKIFK
FvYTH4	AKFFIIKSYSEDDVHKSVKYNVWASTPNGNKKLDAAYQEAQEIATGCPVFLLFSVNASGQFVGLAEMVGPVDFEKNVEYWQQD<br>KWTGCFPVKWHIVKDVPNSLLKHITLENNENKPVTNSRDTQEVKLDEGLKLIKLFK
FvYTH5	AKFFIIKSFSEDNVHKSIKYSVWASTPHGNKKLDAAYHEAKRIKCSCPIFLLFSVNASGQFCGVAEMVGPVDFEKDADYWQQDR<br>WNGQFPVQWHIIKDVPNIRFRHILLENNDNKPVTHSRDCQEVNLNQGIELLKIFN
FvYTH6	AKFFVIKSYSEDDVHKSVKYNVWSSTPHGNKKLSSAYEDAQRIAAGNPGGCPIFLFFSVNASGQFCGMAEMVGPVDFNKDMDF

	WQQDKWSGSFPVIWHIVKDVPNTSVRHIVLENNENKPVTNSRDTQEIMYKKGLEMLKIFK
FvYTH7	TRYFIIKSLSHENIQLSIEKGIWATQVMNEPILEDAFHKSGKVILIFSVNMSGFFQGYAQMMSAVGWRRDNVWSQGSSRSNPWG RSFKVKWLRLNDLPFQQTLHLKNPLNEFKPVKISRDCQELSPDVGEALCELLD
FvYTH8	AKFYVIKSYSEDDVHKSIKYDVWASTPNGNKKLDAAFRDAEAKSSETGKRCPIFLFFSVNGSGQFVGLAEMIGEVDFNKDMDFW QVDKWSGFFPVNWHVVKDIPNTQLRHIILENNDNRPVTFTRDTQEIGLKQGLEMLNIFK
FvYTH9	AKFFIIKSYSEDNVHKSIKYGVWASTPNGNRKLDAAYHEAKEKHDSCPIFLLFSVNASAQFCGVAEMVGPVDFDKSVDYWQQDK WSGQFPVKWHVIKDVPNSQFRHIVLENNDNKPVTNSRDTQEVKLEQGIEMLNIFK

Note: At:*Arabidopsis*; Os: Oryza sativa L.; Fv: *Fragaria vesca L*.

Supplementary Table S5. The identification of *cis*-elements in the promoters of *FvYTH* genes.

Response Types	Responses involved	Cis-elements	FvYTH 1	FvYTH 2	FvYTH 3	FvYTH 4	FvYTH 5	FvYTH 6	FvYTH 7	FvYTH 8	FvYTH 9
hormone	auxin-responsive element	AuxRR-core/ TGA-element	0	0	0	0	1	2	1	0	0
	salicylic acid responsiveness	TCA element	1	2	0	0	0	1	4	0	2
	MeJA-responsiveness	TGACG-motif	1	0	2	0	0	2	1	8	2
	abscisic acid responsiveness	ABRE	2	2	3	5	0	3	4	4	3
	gibberellin-responsiveness	P-box/TATC-box	0	3	1	1	0	2	1	0	1
	ethylene-responsive element	ERE	0	0	1	1	1	0	2	0	0
	total		4	7	7	7	2	10	13	12	8
stress response	low-temperature responsiveness	LTR	0	0	0	2	2	1	0	1	0
	anaerobic induction	ARE	2	5	3	0	2	0	3	2	4
	activation by heat shock, osmotic stress, low pH, nutrient starvation	STRE	3	2	0	6	1	2	5	2	3
	wound-responsive element	WUN-motif	1	0	2	1	0	0	2	2	0
	dehydration- responsive element	DRE	2	0	0	0	1	0	0	2	0
	wound inducibility	WRE3	1	0	0	0	1	0	0	1	0
	wounding and pathogen responsievness	W box	2	0	1	2	0	1	2	0	1
	defense and stress responsiveness	TC-rich repeats	0	1	0	0	1	0	1	0	1
	stress-inducible	TCA	0	0	1	1	2	1	0	0	0
	total		11	8	7	12	10	5	13	10	9
development	meristem expression	CAT-box	2	0	1	0	2	1	1	0	0
	endosperm expression	GCN4_motif	1	0	0	0	0	0	0	0	0
	circadian control	circadian	0	2	0	0	0	0	0	0	0
	zein metabolism regulation	O2-site	0	2	1	0	0	0	0	0	0
	meristem specific activation	CCGTCC-box	0	1	0	0	1	0	0	1	0

	seed-specific regulation	RY-element	0	0	1	0	0	0	0	0	0
	palisade mesophyll cells	HD-Zip 1	0	0	0	0	1	0	0	0	0
	total		3	5	3	0	4	1	1	1	0
transcription factors binding site	Myc-binding site	MYC	4	1	3	4	1	2	4	0	6
	Myb-binding site	MYB	2	7	4	5	6	3	3	2	3
	ATBP-1 binding site	AT-rich element	0	0	1	1	1	0	0	0	0
	MYB binding site involved in drought-inducibility	MBS	1	0	0	1	0	0	0	1	1
	MYB binding site involved in light responsiveness	MRE	1	0	0	0	1	0	0	2	0
	MYB binding site involved in flavonoid biosynthetic genes regulation	MBSI	0	0	0	0	0	1	0	0	0
	total		8	8	8	11	9	6	7	5	10
light	light responsiveness		6	10	9	5	3	9	11	13	12

Supplementary Table S6. The CDS sequences of *FvYTH* genes.

Gene ID	Gene Name	Sequences
FvH4_3g09980	FvYTH1	ATGGAGGACCCCGACGGAGTCCTCAACTTCGACTTCGAGGGCGGCCTCGACTCCGCCGCCGTCTCCGCGC<br>CCACCCACACCGGCCTGGCCTCCAGCGCCCCGATCCAGTCCGACTCGTTCGCCTCCCAGCCCAAAAACCA<br>GGCCGCCCCGGCGCCGCAGCCCGACCCAAATGTCAACCCGTCGGGGCGGAAGAGCTTCCGGCAAACGGTG<br>TGTCGTCACTGGCTCCGGAGCCTCTGTATGAAAGGCGAGGCCTGCGGGTTCCTTCATCAATACGACAAGT<br>CGCGGATGCCGGTGTGCCGGTTCTTCCGTATGTACGGCGAGTGTAGGGAGCAGGATTGCGTGTATAAGCA<br>TACCAACGAGGACATCAAGGAGTGTAATATGTACAAGTTAGGTTTTTGTCCAAATGGTCCTGATTGCCGG<br>TATAGACATGCAAAGCTTCCTGGACCTCCACCCCCTGTGGAAGAAGTCCTTCAGAAGATCCAGCATTTGA<br>ATTCGTACAATTACAACAACTCAAATAAGTTCTCCCAACCACGGAATGGTGGTTTTCCCCAACAACATGA<br>TAGATCTCAGCCCGCACAAGTCACCAATTCATTCAATCAAGTGGTTGTAAGACCTTCAGCAGCAGAGTCT<br>GCTAATGTCCAGCAGCCACAGCAATTTCAGCAAACCCAACAACCGGTTGCCCAGACTCAGGCACAAAGTG<br>TTCCCAATGGCCTGGCTAGTCAGGCAAATAGAGCCGCACTGCCCTTGCCTCAAGGAATATCTAGGTATTT<br>TATTGTTAAAAGTTGCAACCGTGAAAATTTGGAATTATCCGTACAACAAGGAGTTTGGGCAACTCAAAGG<br>AGCAATGAATCCAAACTCAATGAAGCTTTCGACTCTGCAGAAAATGTCATATTAATTTTCTCAGTCAACC<br>GGACTCGACATTTTCAGGGTTGTGCAAAGATGATGTCCAGAATTGGTGGGTCTGTTAGTGGAGGGAACTG<br>GAAATATGCACATGGAACTGCACATTATGGCCGTAATTTCTCTGTCAAATGGTTAAAGCTATGTGAACTT<br>TCCTTCCACAAAACTCGTCACTTGAGGAACCCATACAATGAGAACTTACCAGTGAAGATAAGTAGAGATT<br>GTCAAGAGCTAGAGCCCTCCATCGGTGAGCAGTTGGCATCCTTGCTTTATCTTGAACCAGATAGTGAACT<br>AATGGCAATCTCCATTGCAGCAGAGTCAAAACGAGAAGAAGAAAAAGCAAAGGGAGTCAATCCAGAGAAT<br>GGTGGTGAGAACCCAGACATTGTCCCATTTGAGGACAATGAAGAAGAGGAAGAGGAAGAAAGTGACGACG<br>AGGAAGACTATCAAGTTCCTGGTGGAGCAATTGAGAACAGAGGAAGGGGTAGAGTCATGTGGCCCCCTCA<br>TATGCCACTAGGAGGAAGGGGTGGCAGACCTATGCCTGGGATGCAGGGGTTCCCTGGAATGATGGGTCCT<br>GATGCAATGCCGTATGGACCAGTTACACCTGATGGATTTGTTATGCCCAATCCTTTTGGTATGGGCGGCC<br>CTCGGGGTTTCAATCCATATGGTCCTAGGTTTTCCGGTGATTTTGGTGGGCCCAATCCTGGCATGATGTT<br>TCGTGGGCGGCCTCCACAACCTGGTGGTATGTTTCCACCTGGTCCTTATGGGATGATGATGGGTCCTGGA<br>CGTGGACCATTTATGGGAGGGATGGGGGTTGGAGGTAATAATCCAGCCCGAGGTGGTCGTCCAGGTGGTA<br>TGCCACCAATGTTTCCCCCACACCCACCATCTCAAAACAATAACCGGTTGCAGAAGAGAGATCCAAGGGG<br>ATCGGGCAATGATCGGAATGAGAGGTATAGTGCAGGATCAGGTCATGGCAAAGAAATGCAGGCTGGTGGA<br>CCAGATGATGAGAACCATTATCAGCATTCATCAAAATCGTACCAGGAAGATTATGGTGCTGGGAACAATG<br>GTAGGAATGATGACAGTGAAAGTGAAGACGAAGCACCGAGGCGGTCAAGGCATGGAGAGGGAAAGAAGAA<br>ACGGCGAGACTCGGAAGGAGATGCTACCTCTGAGCACTAG
FvH4_3g13840	FvYTH2	ATGGCTGCTCCTCAATTTGAACAACACAATCCTCATCCTCATCAGACAACTCCCAAACAAGAAGAAGGGA<br>AAGAAAAGGAAGCAAAACCAGATATTCAATCCACAGAGTCCTTCCAATCCAATCTGGCTCTTAGCAAGGA<br>TGGAAGCTCATCTGATGCAGCATCATGTATATCAACTGCAGGAGATACAACTGCTAGTGTGAAAAAAGGT<br>GATGCAGATGTAGATGCAGATTATGAATCCATGGCTTCCTATCCAACCACCGCCTATTATGGATACAGCT<br>ATCCAGGTAGTTATGATATGGAAAACCAAGGATACTATGTCGGTGGAAATACTGGGATGGAAGCTCAATA<br>CCCTGTCATGCAAGCAGATAATGGCTCTTTTGTCTACTTGATGCCAGGATTTCAGCCTGGCTATGATCCT

TACATGCAGTACCTGCCCATGACTACAATCAGTAGTGATGGACAATATGTTGGTCAACAGATGTATTCAT
CGATCAGCCCCATATACCAATCTCCAATCAACTCCCCTGGATATAATATTCCCTCTCCTCTTCCTTATGG
GGAGTTAAGTCCAACACCCTACTTGTGGGACCCATCTCTTGTCGGAGATGGAACATATGGAAATGGCTAC
AATGGAGTTTTAGAAACACCGAATTCTAAATCTAATTTCTCATCCCCTAGTCACACTCGTGCTCCATTCT
CTAAATCTGATACACCACCTTCAGGCAACACTCCTTTAGAGACTAAGAACTTGGGTTATGGTGCACGAAA
TCAGCCAAAACCAGTGAACAAGACATCACTGCATGCTCCGGCTGTCCAATCGGAAGCAGCAGCGAAAGGT
TACTATTCTGTTGCAAAGATCCCAGTCTATAACCAAGGGACAAGTGGTTTGCTGTATCCAAACAACTCGT
TAAAAGCAAATCCAAAGAGTTGGGGTGGTCCTGAGAAGGTGAAAACAAGAAGCAAGGCTAATAGTGTCAG
AGATATCAATTTGTTTAGCGAGAAAAACCACGGTCCCAGAACAACCAATCCTAAGGGCACCATAGTCTCC
GCAGCTGACAAGGAGAATGAAAAATGTGACGGCACAAATTCTATTATCAGGAAGGATCAATATAACCTTC
CTGACTTTCCGACCAAATATGAGCAGGCACTGTTCTTTGTCATCAAATCTTACAGTGAAGATGATATTCA
TAAGAGCATCAAGTATAATGTATGGGCTAGTACTCCTAATGGCAATAGGAGGCTGGACAATGCTTATCAA
GATGCACAAGAGAAGGCGGCACATAAAGGCTGCAAATGTCCTGTGTTCCTCTTCTTTTCGGTTAATGCAA
GCGGTCAGTTCTGTGGAGTAGCTGAAATGGTTGGCCGTGTTGATTTTAATAAGAACATGGAATTCTGGCA
ACAGGATAAGTGGAATGGTTATTTCCCAGTCAAGTGGCATATCATAAAAGATGTTCCAAATCCCCAGTTA
CGACATATAATACTTGAGAATAATGAAAACAAGCCTGTGACCAATAGTCGAGACACGCAAGAGGTTAAAT
TCCTTCAGGGTGTTGAAATGTTGAACATTTTCAAAAATCATTTGTCAAAGACATCGATATTGGATGACTT
TGATTTCTATGAAAGCCGTCAGAAAGTGATGCAAGAGAAGAGGATCAGGCAGTCCACTCCTTACTACGAT
ATGCAGGTTTGCTATTGCTCTTCTTTCATTCTTTTCGGTAACACTTGCATAGTTTCTTTTACTGGCATCT
CCAGTCACAATCATTTAAGGAACATATAG

FvH4_3g45840 FvYTH3 ATGAACAAAACCCTCTTTTTCCAAAAACCCAAACCCTTGGCGGCTGTTAGACCCGTTGTCTCTCTCGCTC
CAAGTCTTCTCTCTCTCTCATCCGTTTTGCTAAACCCCCCAATTTGGGATTTTAGGGATTTGAGGCTGAC
TTTGGAGTTTCGAGCGAGGGCTTCTAGAAGATCCAGCTACCAATTCTCGGCCATGGCCACCGTTGCTCCT
CCCGCAGATCAAGCAGCAGATTTGCTCCAGAAGCTGTCAATAGATTCGCAGACCAAGACTCTGGAAATTC
CAGAGCCTACAAAGAAGCCTTCTGTTAACCAATACGGGTCTATTGATTCTGGCAATGCTGTGAATGGTCA
GATCCCATCTGATCGCTCTGTGACTCCACTGTCGACTGACTTCACGGATCAATCTTTCTCCTATCTCCCA
AATGGTTATCCATCTGCAGCCTATTACTATGGAGGCTATGATGGGGCTGGCGACTGGGATGAGTACTCGA
GATATGTGAATCCAGAGGGAGTAGATATGACTTCGGGGGTTTATGGAGATAACGGGTCTCTTATGTACCA
CCATGGTTATGGGTATGCACCGTATGGGCCTTATTCACCAGCAGCTTCCCCTGTTCCAACTATGGGAAAT
GATGGTCAGTTATACGGGCCTCAACATTACCAGTACCCGCCCTATTTCCAGCCGCTGACTCCAACCAGTG
GGCCATACACTCCCAGTCCTGTAGCCCCTCAGAATGAGGTATCAACTTCCTTAGGTGCTGACCAAAAGCC
TCTGCCAGTGGAAACAGCTAATGGAATTTCTAATGGCATTGCAAATAATGCAAGTGTGAAAGGAAGTAAT
GGTTCAGCTCCCCTATCGACGTATCAAACCACGTCTTTCAACTCCAATGGTTCATATGGACGAGGTGCTT
TGCCTGCACGTGTTCCTACTCCTGGTTACCAGGACCCAAGATATGGTTACGACACAGTACGGTCTCCAAT
CCCCTGGTTAGATGCCCCACTTTTTTCAGATGGGCAGCATAGACCTATGGCAAATACACCCATTACTTCA
AACTCGAATGGCAATAGAATTCCATCTTTGAGGAACCAGAACTACCGTCCAAATTCGCACTACATGGGTT
TGCACCACCCCAGACCGCTGTCAGGAATAGGTACAGCTCAAGGGTTTATGAATAGCAGCATGTACCCAAG
CAAGCTATTTGGCCAGTATGGAAACACAGTCAGATCTGGTCTGGGATATGGGTCTCATGGTTATGATTCG

CGAACCAGTGGACGCACATGGCTGGCTGTTGACAACAAGTATAAACAACCCAGGGGGCGTAATGGTGGTT
ACTATGGATATGGTAATGAGAACTCGGACGGTTTAAATGAGCTCAACAGAGGACCTCGGGCAAGAGGCTC
AAAGAACCAAAAGAGTTTTACCCCTAGTGCATTAGCTATCAAGGGGCAGAATGTGCCAGCAAGTCTTAGT
CATGATGAGGAAAAGGATACAAGTACTGTTCCAGACAGAGAGCAATACAATAAAGCAGATTTTCCAGAGG
ATTATACTGATGCCAAATTCTTTATTATCAAGTCATATAGTGAGGATGATGTCCATAAGAGCATCAAGTA
TAATGTTTGGGCCAGTACACCAAATGGCAACAAGAAGCTTCATGCAGCATACCAGGAGGCTCAGGAGAAG
ACTGGTGGCTGCCCTGTATTTCTTTTTTTCTCGGTCAATACCAGTGGGCAATTTGTCGGCCTCGCTGAGA
TGTTGGGGCCTGTTGATTTCAACAAGAATTTGGAGTACTGGCAGCAAGACAAATGGAATGGCTGTTTCTC
TGTCAAGTGGCATTTTGTTAAAGACGTTCCTAACAGTTTGTTGAAGCATATTACTCTTGAAAATAATGAG
AACAAACCTGTGACTAATAGTAGGGACACTCAGGAGGTTAAGTTGGAACCAGGGCTTAAAATGATCAAAA
TATTCAAGGAGCATTCAAGTAAAACATGCATTTTGGATGACTTTGGATTTTATGAGTCCCGCCAGAAGAC
AATCCAGGAGAAGAAGGCCAAGCAACAGCAGCAGTTTCAGAAGCAGCAGGTATGGGAAGGAAAAACTCTT
GATGAGAAGAAAGAGGTAGTGGCAAATGGACAAGTGAAAACACAAAATTCATCGGATGTTCCCTCTGAGA
TAAACAAGGAATCTATCCCAACAGTGCATGCCAGCGAGGAGCTGAAACTTGCAGAAAATGGATCAATTGC
GACTGGCGAAGCCCCAAAGGGTGCTAAACCGGTTGTGGCTGAGAAGAGGGCTGTAGGAAATGGGGTTGCA
AACGGTTGCTAG

FvH4_3g45841 FvYTH4

ATGGCCACTGTCGCTTCACTCTCGCCTCCTGCGGATCAAGCTTCAGACTTGCTGCAGAATTTGTCGTTGG
ATTCTCAGGCTAAGGATGTCAAGATTTCTGAGCCCCCCAAGAAGGCCAATGCTGTGACTGGAAAGCTCCA
GTCAACTGACCGGACTAGGTCTACTACCCCATCACTCAAGGATTCAACCATGCCCTATCACCCTAATGGT
TACTCCTCTTCTGCTTATTACTATGGAGGCTATGGTGGACTTGAGTGGGGCAACAATATGAATTCTGTTG
TCTATGGTTATGGCTATGCACCCTACAACACATATTCAACAACAGGGTCTCCAACTGTGGGAAGTGGTCA
GCTATATGAACAGCAGTATCAATATCCTCCCTATTTTAAGCAGCTGGCTCCTAAAACTGGTTCACCCTAC
ATGCCTAATTCAGCTGTTCCTTCCAATGGTGGTGTTTCCACCATTAAGGATGCTGACCAAAAGTCCTCAC
CAGTAGGAACAGCTAATGGGATATCCAACAGTACTTTGACCAGTGTGGGGCTGAAGGAAAATAATCTTTC
AGACTTCTCCAAATATCAAAATTCCTCTTTGAATGCAAAGGGTTCCTATGAGGGATACACTGATGCCATA
TATGGTTTTGATGGTTTGATGTCTCCCATACCATGGTTAGAAGGGTCAACGTTTTCAAATGGACAGGCTA
AGGGGACTGGATATACAGGCAACAGAACACTATCTTCTAAGAATCAGATTTCACGACCAAGCTCTCCATA
CATGGGTTTGCTGCCAAGGTTGGGGTATGGTTTGGATACATCTCGTGGACTTAGTGGGATGTATAGCAAC
GATTTATATGGTCATAACTGGAACACATATGGACTTGGTATGGGGTTTGGATCTAATACGTTTGGTATGT
CAAGAAATGGATATGGGTGGACCTCTGTTCAAAACAAGTTTAAACCTAGGGGGCGAGGAAGCAACTTTCG
CTATGGTAACCAGGACATAGATGGGTTGGAAGAGTTGAACAAAGGACCTAGAGGTAAGAGTTATAAAGCT
GGGGTCAATGTCGGGTTGAATAAAGCTAATGATGAGAAAGATACAACTAGCGCCGTTCCAGAGGGGGAGC
AGCGCTCAGGAGCAGATATGCTTGATAATTACTCAGATGCCAAGTTCTTCATTATAAAGTCATATAGTGA
GGATGATGTTCATAAGAGTGTTAAGTATAATGTCTGGGCCAGCACACCAAACGGAAACAAGAAACTTGAT
GCAGCATACCAGGAGGCTCAGGAGATAGCTACTGGCTGCCCTGTATTTCTGCTCTTCTCGGTAAATGCTA
GTGGCCAATTCGTTGGCCTAGCAGAGATGGTAGGGCCAGTTGATTTTGAAAAGAATGTGGAGTATTGGCA
ACAAGACAAGTGGACTGGTTGTTTTCCTGTTAAGTGGCACATTGTAAAGGATGTACCCAACAGTTTGCTA
AAGCACATTACCCTTGAAAACAATGAAAACAAACCTGTCACAAACAGTAGGGACACTCAAGAGGTCAAGT

TAGATGAGGGGCTTAAATTGATCAAACTATTCAAGGATCATTCCAGTAAAACATGCCTTTTGGATGATTT
TGAGTTTTATGAAGATCGCCAGAAGACAATTGAGGAAGTTAAAGCAAAGCGGCAGATCCGGAAGAAGAAG
GTATTGGAGGAGAAGCCCACTGATGGGGTAGAAGAGGCACTAGTCAAATCACCAGAGTCATTGGGAACTA
CATTGGATGTGAAGGAACCAACATCAGTTGATCAGAGTGATGAACAAGTGAAGCCTTTGGAGAATGGAAC
CGTTGCAGAAGCTGGAGATGGCCTAAAGAGTCCACAATCAGTTGTTGAAGAGTCCAAGGTGGCTGAAACA
AATTAG

FvH4_4g00190 FvYTH5 ATGGACGACCAAGATCGAATCGTCTCCACCGGAGAGAGGCCTGTTAAGCCAGATGTCTTGAAAGAGCAGC
CAGCTTCGCCCAAAGATGAGAGGATAGCTTCAGCAAATCCTTCTCCGGCTGCACTCATAACAGGGCCTTC
ACACAATGCCACAGAACAACCCAAGTTTTCAGATGGTGGTGGAGAACATAGCTCCACCCATCCACTCAAT
TTGTATGGGTCCAACGAACCGAGTATGTCTTATAGTGGTTATGGAAGTAGCACTGGTACTTGGGATGGAT
ATCCTCAGTATCTCAATACTGATGGAATGCATATTGTATCACCGGTCATGTACAGTGATAATCCGTCTCT
TCTGTATCACTCTGGTTATGGTTTCAATCCCGAAATTGCATATGGACAATATTCACCAGTCACTACACCT
TTGGCTTCTTATTTCGTAGATGGCCAACTGTATTCTCCACAACAGGTCCCCTTCTCTCCATCTTATTACC
CACAACCGTCTCCTCCAAGTCTGTCTCCTATTACTTCAGCTTTTCCATTGACGCCGACTGAGCTGATGAC
CTCAGAGAGTAGCAGCACTGACAACATGGCCTTTGGGCCAGGATCAGGTTACTTTGTAAATTTTAGATCA
TTTAGTGGAGCAGATCTGTCTGGAAATCTTGGTTCGAGCCCTTTAGCATCTCCAGGGATTTATCCTCAGC
CTATGGGCATACTTGGATCTTATGAACAGAGTGTTGGGCAGATTTCTCATCCACAGAGGTCAATGCATGG
ATATGGGCTGGTATCTAGTTCCTTTACAGGGCGCTACCCACAGAATACTTCTCATCAGGGCTCCAACTTT
GGTGGTCCTTCCCTTTCTTACTCGTTTGGTAATGACCCGAGCCGACTGACTGTTGACAAGGGTAGAAGAA
GAGAGAGGGACTGGGATTCAGTTTCTGTTTTCAGTAATTCACATGATATCTCCAATGACCGCAATCGAGG
ACCGAGGGCTTCAAAGCTGAAGGACAAGAGCGTTTCTGACCAGGGCTCTTCATCTAGTGCCAGGAAAATG
GATTTATCTGCTTCTGGAATCAACCTTGACTCAATTAACCGGCAGGATTTTGTCACAGATTACAAGGAGG
CAAAGTTCTTTATCATTAAGTCTTTCAGTGAGGATAATGTTCATAAAAGTATTAAATACAGTGTTTGGGC
CAGCACTCCCCATGGAAACAAAAAGCTTGATGCTGCTTATCATGAGGCGAAGAGGATAAAATGCAGCTGT
CCAATCTTCCTCTTATTTTCGGTTAATGCTAGCGGACAGTTTTGCGGAGTAGCCGAAATGGTTGGACCAG
TAGATTTTGAAAAAGATGCTGATTACTGGCAGCAAGATAGGTGGAATGGCCAATTTCCAGTTCAGTGGCA
CATTATTAAGGATGTTCCTAACATTCGCTTCCGTCACATTCTACTAGAAAATAATGATAATAAGCCTGTC
ACTCACAGCCGAGATTGTCAGGAGGTGAATCTGAACCAAGGAATTGAGCTATTGAAAATTTTCAATGATC
ATGATGCACGGACATCTATAGTTGATGATTTTGAATTTTATGATGACCGCGAAAGGTCCTTGAAGGAAAG
GAAAGCCAGACAACAGGCTTGTGCAACTAAAGATGCCTCTGATTCACTTGCTCTTGAGACTGTTAAGGAA
ATTTCTAATCTTTCCGATCAAGCCTTGGAGACTGTTAAGGAAAGGAAAGCCAGACAACAAGCTTGTGCAA
CTAAAGATGCCTCTGATTCACTTGCTCTTGAGACTGTTAAGGAAATTTCTAATATTTCCGATCAAGCCTT
GGAGATGAAAGGAAGTAGTGGCAAAGAATTTGCCGAAACTGAACATGAGAGTAGCTCCAAAACGGATTCG
GTTGTATATGAACATGATGCTGTACAGCAAATATCTGATAGTTTATCTCAAGTACTGCAGTTGGAAGAGA
GTGACAAAGAGTTAGTACCATCATCTGAAAGAGGTGTCACACCTGACAGTGAGTTTGTTGATGAGAAGGT
AACAGTTGTATTAACCTAA

FvH4_4g21030 FvYTH6 ATGTATAATGTTCCTGAACATGCAAATAACGAGGTTTATATGATCCAAGGGATGGAACCTAATATGCATT
TCGCTGCTCCCTTTGAACAAGTTGATGCCATGTACAATGAAGGAGCCCCTGAGTATGTTGTTGATCAGGG

CATGTATTATCCAACCGCCAACAACTATGGGTACTACGGTACAGGATTTGAATCACCCGGTGAATGGGAG
GACCAACGTAGGATTTTTGGTGTAGATGGTCCAGATGTCCAGTACACGGGTCCGCAAAATGAAAGTTTGC
CTTATGCATATTATCCACCTGGCTATGGATATGCACAGACTTCATACAACCCTTACAACCCTTACATACC
TGGTGCTATGATAGGTGTTGATGGCCAACAGTATTATACTATCCCCTCTTATCAGAACTCTGTCTCTTCA
CCCGCTTATGTCCCTTTTGTTCAGCCGGATGTCATTCCCAACAGTTCAGCTGAATCTTTGTATGATACTG
GATCATCTATTAACAGACCTGGTGGAAGAGGCCTTCAATATAATTTGAACTCCGCTTCTGGAGCCTTCTC
TAACACTTCTAAGACGTCTTCTAATCAGATGAGCCCCTTGACTAGGTTATTAGAAAGGCCAAGAGGCAAT
GTTGGACCAAACAAGCAAGCTGTAACTCATGGTAGTGTTTCTTCTGGTCGTTTTCCGAACCCTACTCACC
AGAGTAGAAGTGCGTCCGGCTCAGTTTCAGCTGTGGATAACCTTTCAAATGGGAAAGTACTGCCTCATCA
CAATCAATTGAAGGTAGCAATCCCTGTTGGCAATGGCTTATCTGATTTTGGGTCGGGTGCACAAGGACGA
GATGCTGTGGCTAAGCCTCGGCCAAAGTTCCATGTTGGCAGGGCCTTGAATGATATACATGGTAATGTAG
AGCCGTTGAGTGAGCAGAATTGTGGTCCTAGAATCAATAGGTTGAAAAATCAATTAGCTGTTAAAGCCTA
CACAACCAAGGCGGGAGATAGTAATGCACAAGGAAATATCATTATTCGCACCGATCAGTATAACAAGGAA
AGTCTCCCAGTTGATTATGCGCATGCAAAATTTTTTGTAATAAAATCATACAGCGAGGATGATGTGCATA
AGAGTGTTAAATACAATGTGTGGTCATCCACACCCCATGGCAACAAGAAACTAAGCAGTGCTTATGAAGA
TGCCCAACGAATAGCTGCAGGCAACCCTGGAGGCTGTCCTATTTTCCTCTTCTTTTCGGTAAATGCAAGT
GGTCAATTCTGTGGCATGGCAGAGATGGTTGGCCCGGTTGACTTCAACAAGGACATGGACTTTTGGCAGC
AAGACAAATGGAGTGGGAGCTTCCCTGTTATTTGGCACATTGTCAAAGATGTGCCCAACACCAGTGTCCG
GCACATTGTATTGGAGAACAATGAGAACAAGCCGGTGACTAATAGCAGGGATACACAAGAGATCATGTAC
AAGAAAGGCTTGGAGATGTTGAAAATATTCAAGAATCACACAATGAAGACCTCATTGCTTGATGACTTTA
TGTACTATGAGGACCGTCAAAAAATCATGCAAGATGAGAGAGGCAGGTATGTTGTTAGAAATTTCGAGAC
CCCATTCTCAGTAACAGCATTAGGTCCCCCACGCAAGGTGAACCCTGTTCGTGAGCTGCCTCGCAGCAAA
GAGGAAAAGGACACCAAACCTAATGATGCAAACAACTTCGAAAAGTCTCTGGTTCCTGTATCTGAGCAGG
TTTCCTCAAACTTAGATGTTAAATGTGCGAGTATTAGGGGTGGTAAATCAGAGGAAAAGACACTTGAAGC
AGAAGATGATGCTGTATCTACCTTAAAAATTGGTTCTCTCACTATTGATCCCAAGTATGTGGAACTTGAT
TCTTCAGCTGGTGCTGCTACTGCTACTTCAAAAAATGTTGATGTTGTCACTGTTGGCTCAATTCCTGTTA
AAGTAAATGGATTTGCTGAATCCTTGGGTACTTTAACAGTTGGAACGATCCCTCTGGATCCTAGAGCACT
AAAACTTGAGAAGGGCAGTTCTCTCAACTAA

FvH4_5g01140 FvYTH7

ATGTCGTGTGATACTGCAAAGGAAAATGCATCTGTAGTGGATTCATCTGTTACTGAAAGGAAAGAAGACA
TGGGGAATTCAGACGGGCCTGAGAGTTCAAGTCACAAAGGTACTGAGGGGGTTGATCCATCTAAGTCAGA
AGAAGTTGGGCACTCTCATGACCGTGTTTGGGATTCACCTGCAAGAAAAAAGGGTAAATTGTATGGTACA
AGATATTTCATCATTAAGAGTTTGTCACATGAGAATATCCAATTATCCATTGAGAAAGGAATTTGGGCTA
CTCAAGTGATGAACGAACCAATTTTGGAAGACGCTTTTCATAAATCTGGTAAAGTAATTTTAATATTCAG
TGTCAACATGAGTGGATTCTTCCAAGGGTATGCACAAATGATGTCTGCTGTTGGATGGAGGCGAGACAAT
GTATGGAGTCAAGGAAGTAGTAGAAGTAATCCCTGGGGGCGCAGTTTTAAGGTCAAATGGCTGCGATTAA
ATGATCTGCCTTTCCAACAAACTCTTCATCTCAAGAATCCATTAAATGAGTTCAAACCTGTTAAAATTAG
CAGAGACTGTCAGGAGTTGTCTCCAGATGTTGGAGAAGCGCTCTGTGAGCTTCTTGATAGGCATAATGAT
CTGAATGGCATGAACAGTATTGACATGGATGATTTTCCTTCTAAAAGGCCCTGCGTAGAGCCTCCATGTT

CAGTAGCGGAAGAAGAGTATAATATGCCCCCAGTGCATCTCTCATGGTCGCAAACTCCCATGCTCTATCC
TTCCATGCTTTATCAACATCAGGCTGAAACAAAGGGATTTCATTTTGCGAATCAGAAATGTAATGAAAGG
CGGCCAGCTAGTGTTTTGACTGAGGATGATTTTCTTGAAATGTCTTATGAAGAATACCTAGAGGTCTATG
GCAGAAGCAGGAAGGAACTGTATCAGCCTGGTTCTACATCACAGAAGTCATCCAGAAGTAAAGTGCATGA
GGATGATATAAACTCAAGTTACGGAAGTAACCGCAGCTGCTCAAGGAAAAGAGCTCATCAGTCATCCCAT
AAGCAATCACAATGTTGA

FvH4_5g30420 FvYTH8

ATGGCCGGTGAGAAGAAGATTGAGAAAGCTGAACCTGTAGCCACTGTGTTGGCATCAGACTCTGTTAACG
GATTAGGAGAGCCGCAACTGGTCTCTGGGAAAGAAGGGAAACCATATGATGCGATCTCCTCCATGACTTC
TTCAAGGGATGCCACCCATAGCATCAAAGGATATAATGGATCCTATACTCAATTGGACGAACATCGCTAT
GCAAATGCTAGTAATTCACATACGGGTGTGCAGTCGGACAATGGTTCAATGGTCTATTATCTTCCCAGCT
ATAATCCTTATGCCCCTGGAACTCTTATGGGTGTCGATGGCCAAGGTGTCGGTCAACAGCAGTATTATTC
TTCATCAGGTTATCTTCAACCACCTGTTTTATATGGTTCAGAAGCTATGCCTTGTTATTCATGGGACACA
ACACTTTCTGGTGATATCTCTACAGCAACAAACTCTGGTTTTGGAAATCTAAAATCTGGACCTGGTGGCA
CAACTCTATCTAGGTCAAGTGGCTACAAAGCAAATGGCAATCTTGCTAGCAAATTTTCCAAGCCTTTCCC
TTCTACACAACCCATCAAATCTTTAAACAAGGTGCCTTATTTAGGTTCTGATTTCTCAGCCGGTCTTTTG
AAGGGATATCCTTCGACTGGAAGATTCTCATCATTCACTAACCAGAAGGGTCTGTTTCCTCTTAATGGTG
AAGCAAACTACAGATCTAATGGGAGGATTCTAAATGTAAACGACAGATTCAAATCAAGGGATAAATTTAA
TAGAAATGAGGATTTTGAATCCTCAACTGAGTTGACACGTGGTCCAAGATCTCGCAACAAGAGTCCTCCT
TCAGACTCGGCAGTTGAGAAGGAAGTATTGGGGTTTACAGTCAGTAGGGATAAATATAACCTACCAGATT
TTCAGACTGATTATGAAAGTGCAAAGTTCTACGTTATTAAGTCATACAGTGAAGATGACGTCCATAAGAG
CATTAAATATGATGTCTGGGCCAGCACTCCAAATGGCAATAAGAAACTGGATGCAGCATTCCGTGATGCT
GAAGCAAAATCTAGTGAAACAGGCAAACGGTGTCCTATCTTCCTCTTTTTCTCGGTGAATGGTAGTGGGC
AGTTTGTAGGCCTAGCCGAGATGATTGGGGAGGTAGATTTTAACAAAGATATGGACTTCTGGCAAGTTGA
CAAGTGGAGTGGGTTCTTCCCAGTTAATTGGCATGTAGTAAAGGACATACCTAACACTCAGTTGCGGCAT
ATTATCCTTGAAAACAATGATAACAGACCTGTAACGTTTACCCGGGACACACAAGAGATTGGACTGAAGC
AAGGTTTGGAAATGCTTAACATATTCAAAAGTTATGAACCCAAAACATCCTTGCTAGATGACTTCAATTT
CTATGAAAATCGAGAAAAGTCTCTACAGGCCAAAAGGGGCAGCAAACCTGCGACTTTAAAGATTGAAACT
AATGATGATGGAGATATCTCTAAGCATATGAATACTGGAGGGGAAGACTTTGAAGCTAAATCGGGTGGGA
TCGGGATGACTTCTGACCGAACATCTCTCATTTCACTTACAAAAAATCTCTCCCTCAATGCTTGA

FvH4_6g36470 FvYTH9

ATGGCGGCAAACCAACCGCAGGGTACAGATCGTACATCTGACTCTTCTAAGTCACTGAGCATACTGATCA
TGGATGCAGAAGAGAAACCTGCTGAACCTGATACCATGAAGGAGCAGACTCTTGTAGCAAAAAATGAGAG
GTCAATTTCTCCTAATCCTTCTCAGGATGCTGGCCCAATAGGTCATCCAAGGGAAGCAGCAGCTCAACCG
GGTTCTTTCGATCACACTGTTTTTCCACCTAATGTGTATTCCCCTCAAGCACAGCAGACCTTCTACTATA
GAGGTTATGAAAATGGAACTGGTGAGTGGGACGAGTATCCTCCATACCTCAATTCGGAAGGTTTGGAGAT
TACTTCTCCTGGTGTCTACAATGAGAATCATCCATCCCTTGTCTATCATTCTGGGTATAGCTACAATCCG
CAAATGCCATATGGGCCCTACTCTCCGGTCACAACACCTATGCCTTCTGGAGGTGGAGATGGACAGTTAT
ATTCCCCCCAGCAATTTCCATTTTCAGGACCACCTTATTATCAGCAGCTAGGACCTCATAGTATGCCATA
CATTACTTCACCAACTCCAGTTTCACAGCCAGATCTCACCACTTTAGTTAGTATTGATCAACAAGGTGAC

AGCATGCTTTTTGGACCAAGACCTGGTTATTCTCCAGTGGGCTCTTATGGTAGAGGCAGTTTTCCTGGAA
ATCCAGGTAGTTTTGGTTATCATGATATGCAGCAAGGGTTTGATGGATTGAGATCCGGAGGACTTTGGTC
TGACTGGTCAAAACCCTCGGACAGGCAGAGGTCATATACTCCCTTGTCGCCAGCAGTATCTCCACAGCCA
ATTGGCCCACTTGGCTCATTTGGGCAGAATGTCGGAATGGGTTCTCAACACCAAAGGTCTATGTATGGTT
TTGGATCTGGCTCAAACTCTTATAACAGAGGCTACATTCCTAGTGGTTTTAATCAGGGCTCTAGCCTTGG
AAGTGGATCCTTTTCCAGTTTTGGAGCCAACAACCGAGGCTTTCTGTCGTTAGAGAATAGCAAGCAGCAT
GTTAGAGGCACCGGTCCAATATGCAGTTGCAATGCTCCCCTTGATATTCTTTGTGAGCAGAACCGAGGAC
CAAGGGCGTCAAAGCCAAAGGCACAACTCATTGCTGATAACTCAGTTGAGAGCACTAAACTAATTACACC
TACAACCAAGGTTCATGATGAAACATACAACCGGCCAGATTTTGTAACAGAGTACAAAGATGCTAAGTTC
TTTATCATCAAATCTTACAGTGAAGATAATGTTCACAAGAGTATCAAATACGGTGTCTGGGCTAGCACAC
CAAATGGTAACAGAAAGTTAGATGCTGCATATCATGAAGCTAAGGAGAAGCATGATAGCTGCCCAATTTT
TCTCTTGTTTTCGGTGAATGCCAGTGCTCAGTTCTGTGGGGTGGCTGAAATGGTTGGACCTGTTGATTTT
GACAAGAGTGTGGATTACTGGCAGCAAGACAAATGGAGTGGGCAGTTCCCTGTCAAGTGGCATGTAATTA
AGGATGTCCCAAATAGTCAGTTCCGTCACATTGTTCTTGAAAATAATGATAACAAGCCAGTGACTAACAG
TCGAGATACTCAAGAGGTAAAACTGGAGCAGGGAATTGAGATGTTAAATATTTTCAAGAATTTTGAAACA
GAAATGTCCATTCTAGATGATTTTGATTTTTATGAAGATCGTCAGAAGGCAATGCAAGAGCGGAAGGCCA
GACAGCAAGCCAGCCTGGTTACTGTAGGTGTAGTCGGGGAAAATGAGCATAGAAGTACCGTTCAAATTCC
CACTGATCTCATCAAGCACATGTCGAAGAGTTTTGCTCAAGTGGTCCGCTTGGATGAGGGTACTAAAGAA
GCTACCGCTGTTTCTGATGGTCCCATCGGGACTAGAGTGAAACTTGAAGATGCAATTCCAGTGTCCGTCT
CCTCTGCTCAGATCAGTTAG
